# Biomimetic Exogenous “Tissue Batteries” as Artificial Power Sources for Implantable Bioelectronic Devices Manufacturing

**DOI:** 10.1002/advs.202307369

**Published:** 2024-01-09

**Authors:** Ouyang Yue, Xuechuan Wang, Long Xie, Zhongxue Bai, Xiaoliang Zou, Xinhua Liu

**Affiliations:** ^1^ College of Bioresources Chemical and Materials Engineering Shaanxi University of Science & Technology Xi'an Shaanxi 710021 China; ^2^ National Demonstration Center for Experimental Light Chemistry Engineering Education Shaanxi University of Science &Technology Xi'an Shaanxi 710021 China; ^3^ College of Chemistry and Chemical Engineering Shaanxi University of Science & Technology Xi'an Shaanxi 710021 China

**Keywords:** artificial tissue batteries, implantable bioelectronic devices, implantable power sources

## Abstract

Implantable bioelectronic devices (IBDs) have gained attention for their capacity to conformably detect physiological and pathological signals and further provide internal therapy. However, traditional power sources integrated into these IBDs possess intricate limitations such as bulkiness, rigidity, and biotoxicity. Recently, artificial “tissue batteries” (ATBs) have diffusely developed as artificial power sources for IBDs manufacturing, enabling comprehensive biological‐activity monitoring, diagnosis, and therapy. ATBs are on‐demand and designed to accommodate the soft and confining curved placement space of organisms, minimizing interface discrepancies, and providing ample power for clinical applications. This review presents the near‐term advancements in ATBs, with a focus on their miniaturization, flexibility, biodegradability, and power density. Furthermore, it delves into material‐screening, structural‐design, and energy density across three distinct categories of TBs, distinguished by power supply strategies. These types encompass innovative energy storage devices (chemical batteries and supercapacitors), power conversion devices that harness power from human‐body (biofuel cells, thermoelectric nanogenerators, bio‐potential devices, piezoelectric harvesters, and triboelectric devices), and energy transfer devices that receive and utilize external energy (radiofrequency‐ultrasound energy harvesters, ultrasound‐induced energy harvesters, and photovoltaic devices). Ultimately, future challenges and prospects emphasize ATBs with the indispensability of bio‐safety, flexibility, and high‐volume energy density as crucial components in long‐term implantable bioelectronic devices.

## Graphical abstract

The progress of exogenous Artificial Tissue Batteries (ATBs) as power sources for implantable bioelectronic devices in terms of miniaturization, flexibility, and high energy density has been expounded. ATBs consist of three types: energy storage devices, energy conversion devices that utilize body energy, and energy transmission devices that receive external energy, providing technical support for biological activity monitoring, diagnosis, and treatment.

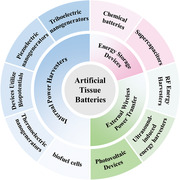

## 1 Introduction

To adapt to intricate microenvironment, organisms rapidly transform different stimuli into nerve impulses in the form of bioelectricity which is transmitted, encoded, and analyzed.^[^ ^1^ ^]^ Dynamic cell membrane permeability modifies the concentration of various ions inside and outside the membrane producing changes in resting or action potentials and eventually resulting in detectable electrical signals.^[^ ^2^ ^]^ The “bio‐cell” in the field of astronautics refers to the collection of electrical energy from bacteria, which provides a much higher efficiency than silicon cells. Thus each cell could be regarded as a biological battery, and macroscopically body organs, such as skin, nerves, heart, blood vessels, bone cartilage, and tendons, consisting of countless cells, are considered endogenous “tissue batteries” (TBs) that maintain a stable physiological state.^[^ ^3^, ^4^ ^]^ Specifically, the vast and complex bioelectricity in the TBs of living organisms is statistically a reflection of physiological activity. Therefore, the signal of bioelectricity can be analyzed to further estimate whether tissues are running normally (electrocardiogram, electroencephalogram, and electromyography).^[^ ^5^, ^6^, ^7^ ^]^ As substantiated, specific intensity and frequency of electricity being delivered to designated sites can affect the physiological state of the tissue, such as restoring the normal rhythm of the heart via a pacemaker.^[^ ^8^ ^]^

The artificial power source that mimics the endogenous electric field in animal tissues and provides power to implantable biomedical electronic devices for biological activity detection, feedback, diagnosis, and treatment is referred to as exogenous artificial “tissue batteries” (ATBs). These ATBs discussed in the article must conform to the soft and limited curved placement space of biological organisms to minimize interface mismatches and provide enough power for implantable bioelectronic devices (IBDs) in the intelligent medicine field, which has attracted increasing global applications in non‐clinical diagnostics and treatments in various fields, including neurology, cardiovascular systems, respiratory system diseases, chronic kidney diseases.^[^ ^9^, ^10^ ^]^ Meanwhile, ATBs have been employed to assemble into novel therapeutic IBDs such as neurostimulators, cardiac defibrillators, ventricular assist devices, and prostheses.^[^ ^11^ ^]^ Further, ATBs within the closed‐loop IBDs drive diagnostic sensors to detect target disease biomarkers and transmit real‐time data to a central processing unit for analysis and later intelligently adjust treatment strategies such as drug release rate and electrical stimulation level.^[^ ^12^ ^]^ Energy‐generating ATBs with characteristics such as flexibility, miniaturization, wireless capability, biodegradability, and controlled lifespan are required by next‐generation personalized IBDs to achieve effective disease prevention, remote diagnosis, real‐time monitoring, and precise intervention.^[^ ^9^, ^13^ ^]^

**Figure 1:**
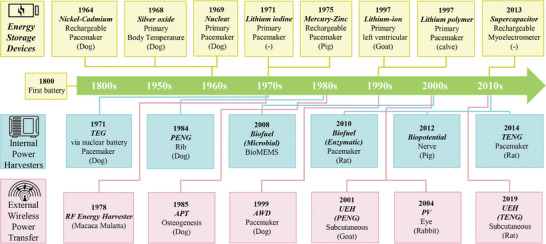
Timeline of important milestones in the development of ATBs for IBDs. Energy storage devices,^[^ ^17^, ^18^, ^19^, ^20^, ^21^, ^22^, ^23^, ^24^, ^25^ ^]^ internal power harvesters,^[^ ^26^, ^27^, ^28^, ^29^, ^30^, ^31^ ^]^ and external wireless power transfer systems.^[^ ^32^, ^33^, ^34^, ^35^, ^36^, ^37^ ^]^ (TEG: thermoelectric nanogenerator, PENG: piezoelectric nanogenerator, TENG: triboelectric nanogenerator, PENG: piezoelectric nanogenerator, RF: Radio‐frequency, APT: Acoustic power transfer, AWD: Auto watch device, PV: photovoltaic, UEH: Ultrasonic Energy Harvesters).

Designing and manufacturing batteries as ATBs remains an enormous challenge. Lithium batteries are considered viable ATBs for powering conventional IBDs, but the bulky size, limited capacity, rigid packaging, and risk of electrolyte leakage hinder long‐term utilization and comfortable implementation clinically because biological organisms are soft, dynamically bendable, and have limited space.^[^ ^14^, ^15^ ^]^ Emerging design principles, flexible materials, and innovative manufacturing techniques were employed in cutting‐edge ATBs to achieve performances like acceptable energy density and power output, ultra‐low self‐discharge rate, soft tissue adaptability, superior biosafety, and physiological and pathological harmonization.^[^ ^16^ ^]^ Since the 1800s (as shown in **Figure** **1**), three main strategies have been utilized: (1) energy storage system improvements (lightweight and flexible batteries, and supercapacitors);^[^ ^17^, ^18^, ^19^, ^20^, ^21^, ^22^, ^23^, ^24^, ^25^ ^]^ (2) internal power harvesters (IPHs) that derive energy from living organisms;^[^ ^26^, ^27^, ^28^, ^29^, ^30^, ^31^ ^]^ and (3) reliable wireless power transfers (WPTs).^[^ ^32^, ^33^, ^34^, ^35^, ^36^, ^37^ ^]^ New materials chemistry and the innovative design of chemical batteries and supercapacitors ensure the capacity of ATBs to match the natural soft characteristics of the organs, research periods are lagging far behind the pace of IBDs.^[^ ^38^, ^39^ ^]^ Thus, the constant and plentiful energy generated by the human tissue, including bioelectricity, thermal energy, mechanical energy, and biochemical energy, is innovatively collected and converted into electricity, which can be applied as a supplementary power source to extend the life of the battery or supercapacitor, or as a stand‐alone power source.^[^ ^40^, ^41^ ^]^ One classical example is the transformation of biomechanical energy from the heart, lungs, and arteries during contraction and diastole into electrical energy using triboelectric/piezoelectric nanogenerator (TENG/PENG).^[^ ^42^, ^43^ ^]^ Biofuel cell (BFC) can collect electricity generated during the oxidation of glucose in living organisms.^[^ ^44^ ^]^ The sufficient recycling of biochemical energy such as temperature (thermoelectric nanogenerator, TEG) and endocochlear potential (EP) dissipated by the human body at all times contributes to the construction of self‐charged and compound‐charged ATBs.^[^ ^45^, ^46^ ^]^ Furthermore, researchers have extensively studied various WPT links and their characteristics, including energy transfer efficiency, transmission depth, and operating frequency, with inductive coupling (IC) being the most established method.^[^ ^47^ ^]^ Although WPT is the preferred ATBs candidate for powering IBDs, there are still some restrained issues that need further investigation and optimization, especially weak coupling, and body‐specific absorption rate suppression.^[^ ^48^ ^]^ Photovoltaic (PV) and ultrasound‐induced energy harvester (UEH) are also gaining interest.^[^ ^49^, ^50^ ^]^ Performance requirements for ATBs used in IBDs vary depending on the device and diagnostic purpose, and high levels of biocompatibility and customizable biodegradability are also essential.^[^ ^51^ ^]^ ATBs for short‐, medium‐ or long‐term biomedical applications require different biodegradability and bioabsorbability.^[^ ^52^ ^]^ Biodegradable ATBs, as a subset of physical transient electronics, operate stably for a while consistent with specific medical needs and then degrade completely in the surrounding biofluid and chemically break down to form benign products that are absorbed or excreted by the body.^[^ ^53^, ^54^ ^]^

The purpose of this review is to provide a comprehensive overview of exogenous ATBs for powering IBDs that have been implanted in living organisms, with a specific focus on compact, flexible, and biodegradable. We will begin by defining and classifying three terms that are commonly used to categorize biomaterials and bioelectronics for ATBs manufacturing: energy storage systems (such as chemical battery and supercapacitor); IPHs (such as BFC, PENG/TENG, TEG, and biopotential device); and WPT systems (such as RF, UEH, and PV). We will then discuss the operational mechanisms, material selection, structural design, energy output, and potential applications for intelligent medicine of these power sources. Furthermore, we will summarize the in vivo performance and assess their potential for future biomedical applications. Finally, we will highlight the dominating challenges and prospects associated with the development of exogenous ATBs.

## 2 Materials and Structural Strategies for ATBs Manufacturing

**Figure 2:**
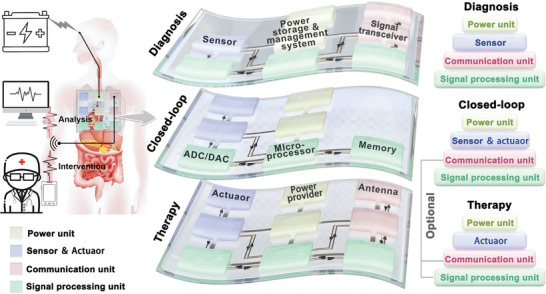
Schematic of functional units for diagnosis, closed‐loop, therapy of IBDs powered by ATBs. (ADC, Analog to Digital Converter; DAC, Digital to Analog Converter)

IBDs that serve as diagnostic, therapeutic, or closed‐loop systems typically consist of various components such as power supply units, energy storage and management systems, sensors, actuators, signal processing units, data memory, and wireless transceivers (**Figure** **2** and **Table** **1**). Treatment tools usually require only a controllable actuator and are considered the simplest systems.^[^ ^55^ ^]^ However, more complex therapeutic procedures, such as time‐controlled dosing or feedback control, require microcontrollers and radio frequency communication modules, which increase the power consumption of the device and necessitate more complex power management circuitry.^[^ ^56^, ^57^ ^]^ Diagnostic devices rely on sensors to collect biological information, microcontrollers for signal processing, wireless communication modules, and data storage to transmit/store processing data.^[^ ^58^ ^]^ In clinical settings, the power output of ATBs for biomedical electronics is specific and limiting.^[^ ^59^ ^]^ Therefore, miniature, biosafe, and biodegradable ATBs with high power density are remade based on the emergent material and architecture designs.

**Table 1:** Summary of IBDs for clinical applications.

Device	Application	Devices	ATBs	Voltage	Power consumption	Disease	Ref.
Diagnostic	Pressure monitoring	Blood pressure monitoring	RF link, TENG, PENG	0.98–4.3 V	8.2 µW–1.2 mW	Hypertension, hypotension	[95, 96, 97, 98]
		Intra‐cranial pressure (ICP) monitoring	RF link, PENG	1–3.3 V	190 nW–75 mW	Traumatic brain injury, brain tumor, hydrocephalus	[99, 100, 101, 102]
		Intraocular pressure (IOP) monitoring	RF, RF for capacitor recharge	0.8–1.1 V	200 nW–200 µW	Glaucoma, ocular hypertension	[103, 104]
		Intra‐abdominal pressure (IAP) monitoring	RF, TENG, Li‐ion battery	1–3.7 V	230 µW–1.5 mW	Abdominal compartment syndrome, aortic aneurysm	[105, 106, 107]
		Bladder pressure monitoring	RF, RF for battery recharge, Li‐MnO_2_ battery	1–3 V	2.3 nW–2.9 µW	Urinary incontinence, Neurogenic bladder dysfunction	[108, 109, 110]
		Cardiac monitoring	Li‐Polymer battery, RF for supercapacitor recharge	1.2–5 V	150 µW–19.95 mW	Arrhythmia, heart attack, ischemic heart failure	[111, 112, 113]
	Temperature monitoring	Core body temperature monitoring	TEG, RF, RF for battery recharge	0.7–3 V	13.33 µW–1 mW	Infection, thermoregulatory disorder	[114, 115, 116]
	Electrical signal monitoring	Electrogastrogram, Electrocardiogram	RF, RF for Li‐ion battery recharge, Li thionyl chloride battery	2.3−3.7V	23 µW−108 mW	Gastroparesis, heart failure, poliomyelitis, myasthenia gravis	[117, 118]
	Medication adherence monitoring	Medication adherence monitoring	Li battery, BFCs	1.85−9 V	1–166 mW	Disease treatment monitoring	[118, 119, 120, 121]
	Drug monitoring	Chemotherapy, anticoagulants	Li battery, BFCs	2.5−3 V	0.22–5.25 mW	Epilepsy, Anticoagulants, Immunosuppression, Cancer	[122]
	Biomarker monitoring	pH telemetry capsule, Glucose, Cortisol	RF, BFCs	0.8–3.3 V	48.5 pW–110 µW	Gastroesophageal reflux, Diabetes, hypoglycemia	[115, 116, 123, 124, 125, 126]
	Imaging	Wireless capsule endoscopy	Li battery	0.85–3 V	35 µW–18 mW	GI bleeding, inflammatory disorder, precancerous tissues	[127, 128, 129]
Therapeutic	Electrical stimulation	Deep brain stimulation	PV, Li battery, BFCs	1−5 V	34.65 µW −1.7 mW	Parkinson's disease, essential tremor	[130, 131, 132, 133]
		Electric nerve stimulation (spinal cord, vagus, and peripheral nerve)	UEH, PV, RF for supercapacitor recharge, Li battery, BFCs	1.5−3.6 V	<1 mW	Diabetic neuropathy, peripheral artery disease, chronic pain relief, sensorineural hearing loss	[134, 135, 136, 137]
		Spinal cord stimulator	Li battery	4.3−50 V	1–10 mW	Chronic neuropathic pain	[138, 139, 140]
		Gastric stimulator	Li‐polymer battery	5–20 V	1–30 mW	Gastroparesis	[141, 142]
	Drug delivery	To GI tract	RF (Inductive coupling) for Li‐ion battery, BFCs	1–3 V	90 µW−7.57 mW	Intestinal or esophageal cancer, inflammatory bowel diseases	[143, 144, 145, 146]
		To subcutaneous space	UEH, PV, and RF for battery recharge	0.4–3.6 V	125 µW–10 mW	Hypertension, anemia	[147, 148, 149]
	Visual prosthesis	Retinal prostheses	PV, Li‐polymer battery	1–2 V	1.2–250 mW	Degenerative retinal diseases retinitis pigmentosa, age‐related macular degeneration	[150, 151, 152, 153]
		Artificial urinary sphincter	PENG for Li‐polymer battery recharge	10 V	200 µW	Rectal neoplasm, Ulcerative colitis	[154]
Closed‐loop	Cardiac assist	Pacemaker	Nuclear battery, Li‐ion battery, TEG, WPT	1.5−5 V	10–500 µW	Arrhythmia, heart attack	[20, 149, 155]
		Cardioverter defibrillator (ICD)	Li‐ion battery	1.2−1.8 V	50 µW–10 mW	Arrhythmia, heart attack	[156]
		Ventricular assist device (VAD)	Li‐ion battery	2–5 V	5–10 W	Cardiomyopathy, hypertensive heart disease	[157, 158, 159]
	Hearing assist	Cochlear implants	PENG, Li battery	0.1–100 mV	100 µW–10 mW	Hearing loss	[9, 160, 161, 162]
	Epilepsy treatment	Neurostimulation	Li battery	<5 V	1.25−15 mW	epilepsy	[163, 164]
	Drug delivery	Blood glucose monitor‐insulin pump	RF, Li‐polymer battery, BFCs	1–2 V	28−70.1 mW	Diabetes mellitus, Parkinson's disease, depression	[143, 165]

### 2.1 Material and Structure for ATBs

The first strategy involves the incorporation of novel flexible materials that possess inherent biocompatibility or biodegradability to accommodate soft tissues and minimize irritation and/or xenobiotic reactions.^[^ ^60^ ^]^ The use of biodegradable materials in device systems is critical compared to traditional implantable materials such as alloys, rubber, and ceramics.^[^ ^61^, ^62^, ^63^ ^]^ Metals (magnesium (Mg), zinc (Zn), tungsten (W), iron (Fe), silver (Ag) molybdenum (Mo)), metal oxides (magnesium oxide (MgO), molybdenum trioxide (MoO_3_), etc.) are often used as electrodes or interconnects.^[^ ^64^, ^65^, ^66^, ^67^, ^68^ ^]^ Polymers (polylactic acid (PLA), polylactic acid‐hydroxyacetic acid copolymer (PLGA), polyvinyl alcohol (PVA), polycaprolactone (PCL), polyanhydride, etc.), natural biopolymers (chitosan, collagen, silk, etc.), and semiconductors (silicon (Si), silicon germanium alloy (SiGe), indium gallium zinc oxide (ITO), etc.) are available as dielectrics, electrolytes, or encapsulants.^[^ ^69^, ^70^, ^71^, ^72^, ^73^, ^74^, ^75^ ^]^ The second strategy is to develop novel structural designs that enable device architectures with compact features and seamless integration of electronic components with biological systems.^[^ ^76^, ^77^, ^78^ ^]^ Photolithography or pre‐strain techniques enable highly tensile structures.^[^ ^79^, ^80^ ^]^ Thus far, the following main structural strategies have been proposed and demonstrated: (1) buckling design, (2) serpentine design, and (3) 3D coiled or stacked structures.^[^ ^77^, ^81^, ^82^ ^]^ The third strategy combines the utilization of flexible materials and tensile structures to more fully enhance the ability of adaptive deformation, electrical performance, stability, miniaturization, and integration.^[^ ^53^, ^77^ ^]^ Potential solutions include the integration of biocompatible high‐energy materials, novel structural solutions, and appropriate ATBs.

### 2.2 Encapsulation

The internal environment of an organism is a highly intricate solution that encompasses various mechanical, electrical, and chemical processes.^[^ ^83^ ^]^ In implanted electronic devices, leakage of elements beyond a certain threshold can result in toxicity, leading to mild inflammation or severe infection.^[^ ^84^ ^]^ To regulate the dissolution rate of the integrated device, an appropriate partial packaging strategy is necessary to prevent excessive local concentration.^[^ ^85^ ^]^ The encapsulation materials must be biocompatible, flexible, and stable, thereby minimizing the risk of leakage and immune reactions.^[^ ^86^ ^]^ Currently, the most commonly used encapsulation materials include polyimide (PI), polydimethylsiloxane (PDMS), poly(tetrafluoroethylene) (PTFE), and chitosan, among others.^[^ ^87^, ^88^, ^89^, ^90^, ^91^, ^92^ ^]^ However, the selection of encapsulation materials needs to be considered from multiple perspectives, as the degradation performance and robustness requirements of the materials vary depending on specific application scenarios, such as short/long‐term or permanent implantation of electronic devices.^[^ ^93^, ^94^ ^]^

## 3 Energy Storage Devices as ATBs

In modern medicine, batteries are crucial components of implanted electronic devices, providing a reliable and convenient source of energy for various bioelectronic devices, including artificial muscles, bioelectronic prostheses, blood glucose monitors, neurostimulators, pacemakers, defibrillators, and cochlear implants.^[^ ^166^, ^167^ ^]^ For IBDs, there are special requirements for ATBs, which must be small, lightweight, long‐lasting, high‐energy‐density, and biosafe with excellent cycling stability to ensure continuous operation in vivo.^[^ ^168^, ^169^ ^]^ Moreover, they must be hermetically sealed to prevent any potential chemical leakage and discharge indication, with excellent compatibility with the soft properties of human tissue.^[^ ^170^, ^171^ ^]^ Conventional chemical batteries such as lithium, zinc‐mercury, and nickel‐cadmium batteries have high energy density and standalone deployment, making them useful in various applications.^[^ ^172^ ^]^ However, new miniaturized biocompatible battery systems are needed to ensure compatibility with the soft properties of human tissue, requiring exploration of new chemistries and reformulation of battery structures.^[^ ^170^, ^173^ ^]^ Supercapacitors have become popular in implantable bioelectronics as important ATBs, as they use ion‐rich liquids or hydrogels as electrolytes to generate electrostatic double‐layer capacitance and/or electrochemical pseudo‐capacitance at the electrodes, which greatly increases the energy density.^[^ ^174^, ^175^ ^]^ They are lightweight, small, mechanically flexible, stable, biocompatible, and biodegradable, making them ideal for energy storage and harvesting applications when high power output or absorption is required.^[^ ^176^, ^177^, ^178^ ^]^ This subsection discusses two types of implantable electrostatic discharge devices as ATBs, including the quantitative properties and limitations of conventional chemical batteries, as well as biocompatible and/or biodegradable novel batteries and supercapacitors. It also covers their material strategies, structural design, and manufacturing options.

### 3.1 Chemical Batteries as ATBs

**Figure 3:**
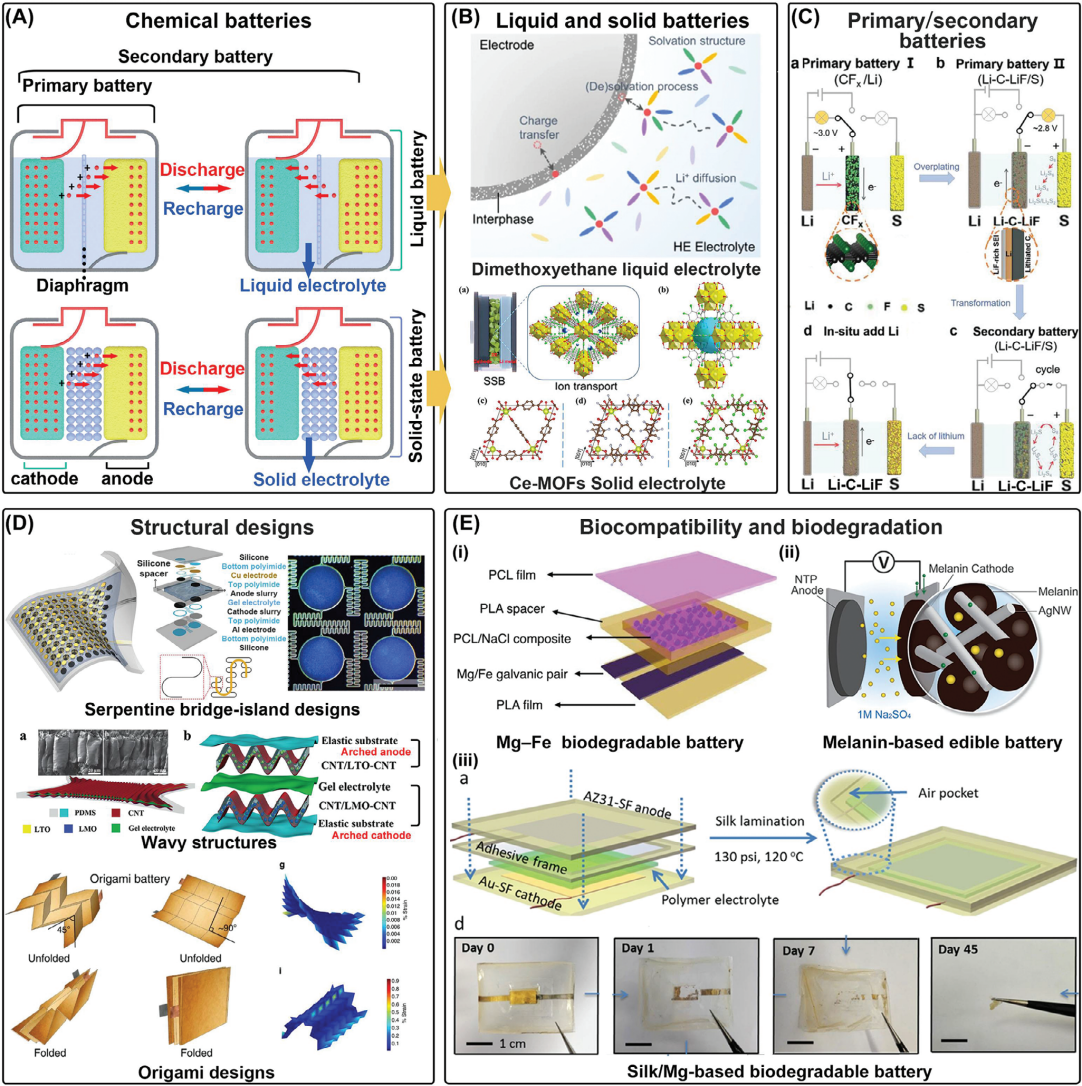
Chemical batteries. A) The power supply mechanism of chemical batteries. B) Liquid and solid batteries. Reproduced with permission.^[^ ^185^ ^]^ Copyright 2018, Springer Nature. Reproduced with permission.^[^ ^186^ ^]^ Copyright 2022, Elsevier. C) Primary and secondary batteries. Reproduced with permission.^[^ ^206^ ^]^ Copyright 2021, Wiley‐VCH. D) The structural designs of chemical batteries. Reproduced with permission.^[^ ^213^ ^]^ Copyright 2013, Springer Nature. Adapted with permission.^[^ ^215^ ^]^ Copyright 2015, Wiley‐VCH. Reproduced with permission.^[^ ^216^ ^]^ Copyright 2014, Springer Nature. E) Biocompatibility and biodegradation of chemical batteries. Reproduced with permission.^[^ ^225^ ^]^ Copyright 2019, Springer Nature. Reproduced with permission.^[^ ^231^ ^]^ Copyright 2015, Wiley‐VCH. Reproduced with permission.^[^ ^223^ ^]^ Adapted 2017, American Chemical Society.

Implantable chemical batteries are available in both rigid and flexible form factors. Traditional rigid chemical batteries have been a reliable power source for implantable electronic devices such as cardiac implantable electronic devices.^[^ ^179^, ^180^ ^]^ The first ATBs, a nickel‐cadmium (NiCd) battery, used for pacemakers was developed and introduced in the late 1950s, and it was first reported in a journal in 1964.^[^ ^18^ ^]^ It provided a significant improvement over the previously used mercury‐zinc batteries, offering longer‐lasting power and better performance for implantable medical devices like pacemakers. Further, both primary (single‐use) and secondary (rechargeable) batteries have been developed that can provide continuous power for IBDs in the human body for up to many years.^[^ ^181^, ^182^ ^]^ **Figure** **3**A illustrates that the positive electrode of a primary battery undergoes an oxidation reaction, losing electrons, while the negative electrode undergoes a reduction reaction, gaining electrons. This electron flow provides electrical energy to the load. However, as the reaction is irreversible, a primary battery cannot be reused once it has consumed all the reactants. In contrast, the chemical reaction in a secondary battery is reversible, allowing it to be charged and discharged multiple times. During charging, the chemical reaction between the positive and negative electrodes is reversed, causing the negative electrode to lose electrons and the positive electrode to gain electrons.^[^ ^183^, ^184^ ^]^ Sufficient capacity is crucial for meeting the power requirements of IBDs, so solid‐state electrolyte batteries have been developed in recent years to increase density. In Figure 3B, a liquid battery has a liquid electrolyte that provides high conductivity and low internal resistance but is prone to leakage and corrosion. In contrast, a solid‐state battery has a solid electrolyte which offers better safety, stability, energy density, and longevity, but has higher manufacturing costs and still faces challenges in conductivity and internal resistance. Additionally, they eliminate the need for separators and other packaging restrictions, enabling more flexible cell structure designs.^[^ ^185^, ^186^ ^]^

#### 3.1.1 Primary and Secondary Batteries

Primary batteries, despite their high cost and limited lifespan, are still widely used due to their high energy density and technical reliability.^[^ ^170^ ^]^ Lithium‐iodine batteries were the main source of power for low‐voltage, microampere current, single‐ and dual‐chamber pacemakers until the early 1970s, although they had limited output power.^[^ ^187^, ^188^ ^]^ Other types of batteries used included zinc‐mercury (Zn‐Hg), nickel‐cadmium (Ni‐Cd), lithium‐silver (Li‐Ag), lithium‐copper sulfide (Li‐CuS), and lithium thionyl chloride (Li‐SOCl_2_).^[^ ^189^, ^190^ ^]^ However, for specific bioelectronic products, batteries need to provide appropriate power levels ranging from microampere to ampere level currents depending on the requirements.^[^ ^191^ ^]^ To meet this need, other lithium anode chemicals were introduced, including lithium‐carbon monofluoride (Li‐CFx), lithium‐manganese dioxide (Li‐MnO_2_), lithium‐silver vanadium oxide/carbon monofluoride blends (Li‐SVO/CFx), and lithium thionyl chloride (Li‐SOCl_2_).^[^ ^192^, ^193^, ^194^, ^195^, ^196^ ^]^ By the beginning of the 21st century, many significant technological advances in implantable medical devices made it necessary for power supplies to provide milliampere currents for data recording, telemetry communications, and programming. This has led to the development of batteries with higher power capabilities, such as lithium‐vanadium pentoxide (Li‐V_2_O_5_), lithium‐silver vanadium oxide (Li‐SVO), lithium‐manganese dioxide (Li‐MnO_2_), and hybrid lithium‐silver vanadium oxide/carbon monofluoride flakes (Li‐SVO/CFx).^[^ ^197^, ^198^, ^199^, ^200^ ^]^

Rechargeable batteries are now able to be charged while implanted in the human body, removing the need for complex replacement procedures. Typically, rechargeable batteries are composed of a carbonaceous anode and a metal oxide cathode, such as lithium (Li), nickel (Ni), aluminum (Al), manganese (Mn), and cobalt (Co)‐based oxides.^[^ ^201^, ^202^, ^203^, ^204^ ^]^ These types of batteries have been instrumental in the development of durable implantable bioelectronics for the treatment of human diseases. However, primary lithium/graphite fluoride (CFx) batteries, with a high energy density of 3725 Wh kg^−1^, have limitations in their application inside the body due to the generation of hazardous waste, such as highly reactive lithium metal and lithium fluoride (LiF).^[^ ^205^ ^]^ To address these limitations, a secondary battery configuration has been developed using fluoride graphite as a reversible lithium anode in a three‐electrode configuration with lithium, fluoride graphite, and sulfur electrodes, mixed with CFx‐Li negative electrodes (Figure 3C). This new rechargeable battery has a high energy density of up to 507.7 Wh kg^−1^ and a cycle life of 2000 h.^[^ ^206^ ^]^ These secondary batteries have a suitable voltage, energy density, low self‐discharge design, and a very small size with sufficient rechargeable capacity, presenting an opportunity for medical batteries. Despite these advancements, secondary batteries have been shelved due to issues such as undercharging, overcharging, overheating, battery rupture, loss of battery capacity, and local tissue heat without medical supervision.^[^ ^207^, ^208^ ^]^ Therefore, additional research and development are necessary to overcome these challenges and fully realize the potential of rechargeable batteries for medical applications.

#### 3.1.2 Structural Designs

Maintaining the stability of stretchable ATBs is crucial for implantable biomedical applications as any disturbance in current output can lead to signal interference in data collection components.^[^ ^209^ ^]^ Although lithium‐ion batteries still dominate the battery market due to their high energy capacity and lightweight, attempts have been made to use intrinsically stretchable materials such as carbon nanotubes or graphene, but these have not been able to compete with conventional active materials.^[^ ^210^ ^]^ Therefore, current research in the construction of stretchable batteries mainly focuses on the structural design of conventional batteries and composites of stretchable materials.^[^ ^211^, ^212^ ^]^ As shown in Figure 3D, advanced structural designs such as serpentine bridge‐island designs,^[^ ^213^, ^214^ ^]^ wavy structures,^[^ ^215^ ^]^ origami designs^[^ ^216^, ^217^ ^]^ have been proposed to simultaneously confer excellent stretchability and energy density for tissue adaptation. designs have demonstrated impressive properties such as up to 1300% linear deformability,^[^ ^216^ ^]^ over 85% capacity retention, and 99.8% coulombic efficiency in 100 cycles.^[^ ^218^ ^]^ However, devices based solely on structural design are too rigid and bulky for full adaptation to the working environment within the human body. Therefore, strategies based on elastic materials are being proposed to achieve miniaturization of such devices with micron‐scale thicknesses.^[^ ^218^, ^219^ ^]^

#### 3.1.3 Biocompatible and Biodegradable Batteries

In addition to stability and energy density, biocompatibility is also a crucial factor in developing ATBs for implantable biomedical applications.^[^ ^170^ ^]^ To achieve biocompatibility, researchers have focused on using biodegradable metals such as magnesium (Mg), zinc (Zn), iron (Fe), tungsten (W), and molybdenum (Mo) as anodes in thin films or sheets. For cathodes, thin film materials such as platinum (Pt), copper (Cu), gold (Au), polypyrrole (PPy), elastic silk, cellulose films, catechol, melanin, polydopamine (PDA) are used.^[^ ^167^, ^220^, ^221^, ^222^, ^223^ ^]^ Solid‐state electrolytes and encapsulation materials made of biodegradable polymers or hydrogels like poly(lactic‐co‐glycolic acid) (PLGA) and alginate, as well as dissolvable oxides (MoO_3_) and polycaprolactone (PCL) films, are used to further enhance biocompatibility (Figure 3E_i_).^[^ ^224^, ^225^ ^]^ Additionally, liquid metal at room temperature can also be used as a cathode material due to its high elasticity, including high stretchability (100%) and recovery (with a discharge performance retention of 98.87%), which is suitable for long‐term effective implantable ATBs.^[^ ^226^, ^227^ ^]^ By optimizing the materials and corresponding structures of ATBs, biocompatibility and biodegradability can be achieved, making them suitable for implantable biomedical applications.

To meet the requirements of temporary implantable medical devices, ATBs must be biodegradable. One such system is the high‐performance primary magnesium‐molybdenum trioxide (Mg‐MoO_3_) battery, which achieved a stable voltage of up to 1.6 V and a power density of 0.27 mW cm^−2^. This battery was shown to be completely biodegradable and highly biocompatible (with a daily allowance of ≈300 mg d^−1^) both in vitro and in vivo.^[^ ^224^ ^]^ Furthermore, the physiological fluid as the electrolyte is a novel and effective design approach that avoids the use of chemical electrolytes. Another biocompatible battery design incorporates a conductive fiber with polydopamine/polypyrrole as the anode and MnO_2_ as the cathode, with body fluid serving as the electrolyte. This battery integrates well with biological tissue when minimally invasively injected into the body and can biodegrade after completing its task, avoiding the need for secondary removal surgery. Despite its biodegradability, this battery can still power a wide range of in vivo biosensors, with a specific capacity of 25.6 mA h g^−1^ and a retention rate of 69.1% after 200 charge/discharge cycles.^[^ ^228^ ^]^ Other studies have shown that physiological liquids provide better battery performance as battery electrolytes than other chemical electrolytes, due to their suitable pH−buffering abilities (Figure 3E_ii_). The biodegradable Mg/Fe battery performed an energy density of 694 Wh kg^−1^ and a total volume of 0.02 cm^3^.^[^ ^229^, ^230^ ^]^ Additionally, melanin‐based sodium‐ion batteries are used as a power source for edible and implantable medical devices, utilizing silver nanowire cathodes and sodium titanium phosphate (NTP, NaTi_2_(PO_4_)_3_) anodes.^[^ ^231^ ^]^ Figure 3E_iii_ illustrates that using fully biocompatible and degradable materials as cathode materials and electrolytes are effective strategies for the preparation of ATBs. Elastic filament cellulose films have also been reported as alternative cathode materials for magnesium‐air batteries, and silk fibroin‐choline nitrate has been developed as a polymer electrolyte that can be decomposed in a concentrated buffered protease solution.^[^ ^223^ ^]^

### 3.2 Supercapacitors as ATBs

**Figure 4:**
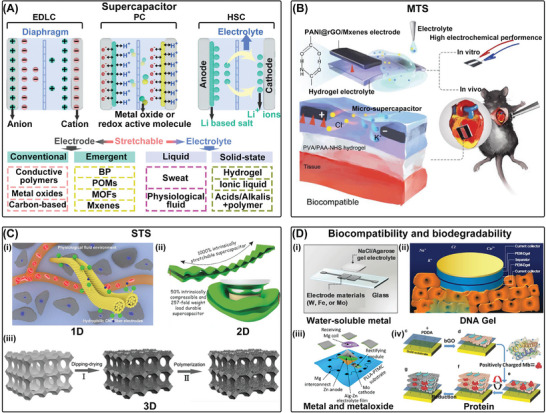
Supercapacitors. A) The power supply mechanism and conventional materials of supercapacitors. B) Materials that stretch (MTS) for flexible supercapacitors. Reproduced with permission.^[^ ^248^ ^]^ Copyright 2021, Wiley‐VCH. C) structures that stretch for flexible supercapacitors. Reproduced with permission.^[^ ^235^ ^]^ Copyright 2017, Elsevier. Adapted with permission.^[^ ^251^ ^]^ Copyright 2017, Wiley‐VCH. Adapted with permission.^[^ ^252^ ^]^ Copyright 2015, Wiley‐VCH. D) Biocompatibility and biodegradability of supercapacitors. Reproduced with permission.^[^ ^234^ ^]^ Copyright 2017, Wiley‐VCH. Adapted with permission.^[^ ^253^ ^]^ Copyright 2013, Springer Nature. Adapted with permission.^[^ ^257^ ^]^ Copyright 2017, Wiley‐VCH. Reproduced with permission.^[^ ^254^ ^]^ Copyright 2023, Advanced Science.

Supercapacitors are promising electrochemical energy storage devices, which offer several advantages over conventional batteries, such as superior power and energy density, cycle durability, rapid dis/charging rates, and a memoryless effect.^[^ ^38^, ^232^ ^]^ Unlike batteries, the charge storage mechanism in supercapacitors depends on ion electroadsorption at the electrode/electrolyte interface (electric double‐layer capacitance, EDLC), reversible Faraday redox reactions, electroadsorption, or embedding processes occurring at or near the electrode interface (pseudocapacitance, PC), or a combination of Faraday and non‐Faraday processes to store charge (hybrid capacitance with a combination of EDLC and PC, HSC).^[^ ^40^, ^233^ ^]^ As shown in **Figure** **4**A, the electric double layer creates capacitance, and the energy is stored by the separation of electrical charges at the electrode/electrolyte interface. When a supercapacitor is connected to a power source, electrons flow from the source to the electrode while positive ions move from the electrolyte to the surface of electrode. These ions are adsorbed on the surface of the electrode, creating a charge layer known as the electric double layer. When the supercapacitor is connected to a load, these ions return to the electrolyte while electrons flow out of the electrode, generating current. Supercapacitors can be seamlessly integrated with other energy harvesting or transfer devices, like TENGs and RFs, to form rapid energy storage and supply systems. They are also suitable for use in various IBDs, including implantable neurostimulators, cardiac pacemakers, biochemical/stress sensors, and electromyographs. However, conventional supercapacitors are often rigid and heavy, and the unstable electrolyte necessitates complex and rigid encapsulation prior to implantation, making them unsuitable for demanding bioelectronic systems.

Two feasible principles have been proposed to achieve conformal attachment of stretchable and flexible supercapacitors as ATBs. The first principle is based on the use of Materials that Stretch (MTS), where inherently stretchable and flexible biomaterials replace every component of conventional energy devices, such as electrodes, electrolytes, spacers, and collectors. While new materials, such as metal nanowires, metal oxides, carbon materials, MXenes, organic polymers, metal‐organic frameworks (MOFs), and polyoxometalates (POMs), have been widely applied in stretchable ATBs, they usually crack and delaminate when subjected to more than 10% strain.^[^ ^54^, ^234^, ^235^, ^236^, ^237^, ^238^, ^239^ ^]^ The second principle is based on Structures that Stretch (STS), which can be categorized into 1D fiber‐like structures (parallel, twisted, and core‐shell fibers), 2D configurations (sandwich, interlocked, and arrays), and 3D spatial structures (kirigami, sponge, and fabric), which can provide outstanding stretchability.^[^ ^240^, ^241^ ^]^ By synergistically combining STS and MTS strategies, stretchable, flexible, and biocompatible ATBs can be seamlessly attached to the tissue. Overall, the proposed strategies provide promising avenues for the development of stretchable supercapacitors that can be safely and effectively integrated with human tissue, paving the way for next‐generation wearable and implantable bioelectronics.

#### 3.2.1 Materials that Stretch (MTS)

Stretchable materials with excellent biocompatibility, known as MTS, have been selected to meet the essential requirements for implantable devices. Various emerging materials such as carbon‐based materials graphene, conductive polymers, ionic liquids, and composite conductive materials are used as electrodes and electrolytes to construct stretchable supercapacitors.^[^ ^242^, ^243^, ^244^, ^245^, ^246^ ^]^ For instance, fiber electrodes for supercapacitors have been prepared using Poly(3,4‐ethylenedioxythiophene): poly(styrene sulfonate) (PEDOT: PSS)/ferritin nanoclusters trapped in multi‐walled carbon nanotubes (MWNTs). This supercapacitor has a surface capacitance (32.9 mF cm^−2^) and a surface energy density (0.82 µWh cm^−2^) in PBS, and biocompatibility allowed it to operate well in mice and maintain a capacitance above 90% after eight days.^[^ ^247^ ^]^ Additionally, Figure 4B shows that a biocompatible micro‐supercapacitor entirely composed of hydrogel materials, including a polyaniline@reduced graphene oxide/Mxenes gel electrode, demonstrated remarkable area capacitance and energy density (45.62 F g^−1^, 4.68 Wh kg^−1^).^[^ ^248^ ^]^ Another promising material, bio‐ionic liquid (BIL), has been shown to have potential applications. The implantable supercapacitor prepared using BIL has a volumetric capacitance of ≈44 µF cm^−3^, cycling stability of up to 10 000 cycles, and an energy density almost as high as that of an implantable battery.^[^ ^249^ ^]^

#### 3.2.2 Structures that Stretch (STS)

A new 1D supercapacitor in Figure 4C_i_ was developed using biocompatible stretchable hydrophilic CNT fibers synthesized continuously, where physiological fluids such as phosphate buffer saline (PBS), serum, and blood were utilized directly as electrolytes. The specific capacitance of the device was 10.4 F cm^−3^ or 20.8 F g^−1^, which was retained at 98.3% after 10000 cycles in PBS.^[^ ^235^ ^]^ Another effective approach for creating stretchable energy devices is the 2D serpentine bridge island design, which involves connecting rigid materials like silicon with stretchable serpentine conductive patterns (via gold bars).^[^ ^250^ ^]^ This serpentine interconnection allows the rigid active “bridge” assembly to take up most of the deformation during stretching, thus preserving the functionality of the entire device. Figure 4C_ii_ shows a novel polyelectrolyte consisting of polyacrylamide hydrogel and vinyl hybrid silica nanoparticles (PAM–VSNPs) for the fabrication of wavy‐pattern supercapacitors, exhibiting a superstretchability of 1000%, 2.6‐fold capacitance enhancement, and 50% compressibility with considerable retention of capability. This prestretching method is extended to biaxial directions so that all in‐plane directions are stretchable.^[^ ^251^ ^]^ Moreover, 3D porous structures have a high surface area/volume ratio, which is essential for increasing energy/power density. Figure 4C_iii_ illustrates a solid‐state supercapacitor with excellent compressibility that was fabricated using a PANI‐SWCNTs sponge electrode prepared by the “dipping and drying” method, and polyvinyl alcohol/sulfuric acid gel electrolyte. The device displayed a high capacitance (216 F g^−1^), power density (1.5 kW kg^−1^), and energy density (8 W h kg^−1^) under 60% strain.^[^ ^252^ ^]^

#### 3.2.3 Biocompatible and Biodegradable Supercapacitors

Natural biomolecules with both biocompatibility and biodegradability have great potential for the development of temporary flexible supercapacitors. Examples of such biomolecules include melanin,^[^ ^239^ ^]^ deoxyribonucleic acid (DNA) hydrogel,^[^ ^253^ ^]^ and ferritin.^[^ ^247^ ^]^ The earliest biodegradable and biocompatible supercapacitor with soluble metal electrodes and agarose gel electrolyte shows a capacitance and power density of 1.6 mF cm^−2^ and 1 mW cm^−2^ (Figure 4D_i_).^[^ ^234^ ^]^ In the case of implantable devices, using physiological fluids as electrolytes is an even better choice. DNA hydrogels can also be used as templates to prepare supercapacitors that exhibit excellent charge/discharge stability in physiological fluids like PBS solution or urine and have little cytotoxicity in cell culture media cycling tests for tissue implantation applications (Figure 4D_ii_).^[^ ^253^ ^]^ However, some of the aforementioned polymeric materials may not be suitable for the simple construction of IBDs that can be reabsorbed in the human body. To address this issue, A capacitor that is fully bioresorbable was created, as illustrated in Figure 4D_iii_. This capacitor comprises a polymer substrate supported by poly(l‐lactic acid) and poly(trimethylene carbonate) (PLLA‐PTMC), biodegradable iron (Zn)/molybdenum sulfide (MoS_2_) nanosheets as anode/cathode, and ion‐crosslinked alginate gel as electrolyte. This capacitor achieves high capacitance (93.5 mF cm^−2^) and output voltage (1.3 V) and can be totally biodegraded and absorbed by rats after completing its mission.^[^ ^254^ ^]^ It is noteworthy that this study, building upon previous research, has increased the output voltage, enhanced flexibility, and provided a reliable wireless charging strategy, overcoming the drawbacks of the need for periodic replacement.^[^ ^255^ ^]^ Further, they developed degradable supercapacitors that provide a high area capacitance (181.86 mF cm^−2^), an energy density (30.56µWh cm^−2^), and a high output voltage (1.6 V).^[^ ^256^ ^]^ Additionally, completely non‐biotoxic protein‐based bioelectrochemical capacitors have been developed that are fully flexible and have high power density comparable to lithium thin film batteries (Figure 4D_iv_).^[^ ^257^ ^]^

**Table** **2** summarizes the recently developed energy storage devices, which have achieved in vitro energy harvesting and can be applied to power IBDs for non‐clinical disease treatment and detection. Implantable supercapacitors offer advantages such as fast charging and discharging, long lifespan, and high power density. However, they exhibit relatively lower energy density and higher costs. In contrast, implantable batteries have higher energy density, cost‐effectiveness, and diversity, but they come with drawbacks like slow charge and discharge rates, limited lifespan, and potential safety risks. When selecting implantable energy storage devices for specific applications in the medical and bioimplant field, a careful balance between the strengths and weaknesses of supercapacitors and batteries is crucial to meet the requirements and ensure safe and reliable performance.

**Table 2:** Summary of energy storage devices for clinical application.

Type Advantages disadvantages	Cathode	Anode	Electrolyte	Application	Recharge and biodegrade	Voltage or areal capacitance	Currents density	Power density	Ref.
**Chemical battery** Stable power supply, small size, high energy density; but limited lifespan, hazardous substances, maintenance required	MnO_2_	Li	LiPF_6_	Pacemaker	No No	6 V	−	−	[258]
	MoO_3_	Mg	Sodium Alginate	IBDs	No Yes	1.6 V	0.3 mA cm^−2^	0.27 mW cm^−2^	[224]
	LiCoO_2_	Graphite	LiPF_6_	Artificial Heart	Yes No	3.7 V	−	148 Wh kg^−1^	[259]
	VOPO_4_	Carbon	Sodium‐Ion	Wearable devices	Yes No	3.75 V	50 mA g^−1^	126 mA h g^−1^	[260]
	Zn film	MnO_2_/reduced graphene oxide (rGO)	Cellulose aerogel‐gelatin	IBDs	Yes Yes	0.85–1.9 V	61.6 mA g^−1^	211.5 mAh g^−1^	[167]
	MnO_2_	Polydopamine (PDA)/polypyrrole	Body fluid	IBDs	Yes Yes	1.2 V	1000 mA g^−1^	25.6 mAh g^−1^	[228]
**Supercapacitor** High energy density, long lifespan, fast charge/discharge, biosafety; But large in size, poor stability, high cost	Polyaniline (PANI)@rGO/Mxenes		Polyacrylamide (PAM)‐alginate	IBDs	Yes No	45.62 F g^−1^	100 mA g^−1^	−	[248]
	MWCNT/rGO		Propylene carbonate‐poly(methyl methacrylate)−1‐butyl‐3‐methylimidazolium bis(trifluoromethylsulfonyl) imide	Wearable devices	Yes No	30.4 mF g^−1^	0.025 mA cm^−2^	393.5 µW cm^−2^	[261]
	Poly(3,4‐ethylenedioxythiopene) on		−	Wearable devices	Yes No	408.4 mF g^−1^	1.6 mA g^−1^	−	[262]
	PDA‐zeolitic imidazolate framework 71 NPs in PAM								
	Polypyrrole/graphene oxide coated nickel		MXene‐based PAM	IBDs	Yes No	53.8–91.5 F g^−1^	0.3–1 A g^−1^	−	[178]
	Anodized molybdenum oxide film (Mo_2_O_3_)		Polyvinyl alcohol (PVA) with ionic salts (Na^+^ and Cl^−^)	Wearable devices	Yes Yes	4.15 mF cm^−2^	0.05 mA cm^−2^	0.8 mW cm^−2^	[263]
	Metal (W, Fe, and Mo)		NaCl/agarose	IBDs	Yes Yes	1.6 mF cm^−2^	0.15 mA cm^−2^	61 µW cm^−2^	[234]
	MoO_3_‐MoS_2_		PVA/phosphate buffer saline	Wearable devices	Yes Yes	181.86 mF m^−2^	0.5 mA cm^−2^	−	[256]

## 4 Internal Power Harvesters (IPHs) as ATBs

The human body generates various forms of energy, including thermal, mechanical, electrical, and chemical energy. These different forms of energy are interconnected and work together to maintain the body's homeostasis and enable its various functions.^[^ ^264^ ^]^ Chemical energy is stored in molecules such as protein glucose, ATP, and fat and is used as a source of fuel for the body's functions. Chemical energy can be harvested from the human body through biofuel cells (BFCs).^[^ ^44^, ^265^ ^]^ BFCs can generate electrical energy by catalyzing the oxidation of biofuels, such as glucose and lactic acid, using biocatalysts at the anode and reducing oxygen at the cathode.^[^ ^266^ ^]^ However, there are technical challenges to overcome such as stability and the requirement for expensive precious metals. Inorganic nanomaterials and nanocomposites have shown promise in addressing these challenges, but more research is needed to develop low‐cost and biocompatible materials.^[^ ^267^ ^]^ Lactic acid is a particularly attractive biofuel due to its higher levels in body fluids, but it has been less studied compared to glucose.^[^ ^268^ ^]^ Thermal energy can be harnessed using thermal energy harvesters that convert heat into electricity through the Seebeck effect. These devices utilize a temperature gradient between two types of conducting materials, P‐type and N‐type.^[^ ^269^ ^]^ Various thermoelectric materials have been studied for human body heat collection, including P‐type materials such as aluminum, gallium, and boron, and N‐type materials such as phosphorus, arsenic, and antimony.^[^ ^270^ ^]^ Some thermoelectric samples have been implanted in experimental animals to generate observable electrical outputs. Some studies have shown that weak potential differences can be generated within the human body, such as between cell membranes and neurons, which can be collected through an energy harvester.^[^ ^271^, ^272^ ^]^ However, these technologies still face many challenges, such as improving energy conversion efficiency and increasing material biocompatibility and stability.^[^ ^273^ ^]^ Mechanical energy generated by muscles, which enables movement, can be collected through mechanical energy harvesters. These devices can convert mechanical energy into electricity using piezoelectric and triboelectric effects.^[^ ^274^ ^]^ Some mechanical energy harvesters have been implanted in experimental animals to produce measurable electrical output for non‐clinical disease detection and treatment.^[^ ^275^ ^]^ Although there are still technical challenges to overcome, such as improving energy conversion efficiency and ensuring material biocompatibility, these methods have the potential to become a new energy source for medical and wearable electronic devices in the future.^[^ ^276^ ^]^

### 4.1 Biofuel Cells (BFCs) as ATBs

**Figure 5:**
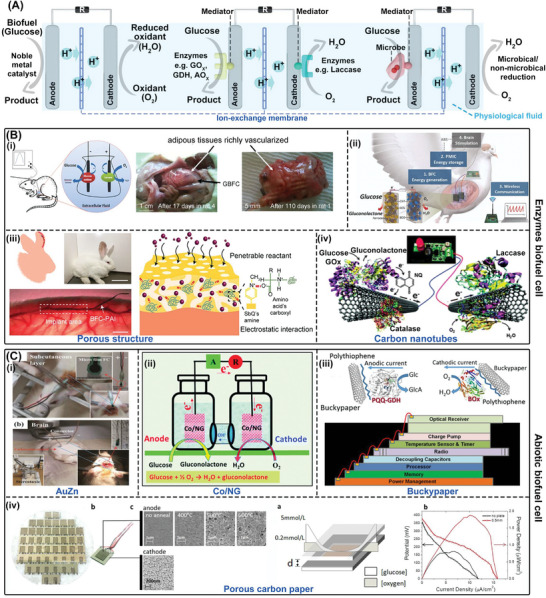
Biofuel cells. A) The power supply mechanism of biofuel cells. B) Enzymes biofuel cells. Adapted with permission.^[^ ^285^ ^]^ Copyright 2013, Springer Nature. Reproduced with permission.^[^ ^133^ ^]^ Copyright 2021, Elsevier. Reproduced with permission.^[^ ^286^ ^]^ Copyright 2021, Wiley‐VCH. Reproduced with permission.^[^ ^287^ ^]^ Copyright 2013, Royal Society of Chemistry. C) Abiotic biofuel cells. Reproduced with permission.^[^ ^298^ ^]^ Copyright 2015, Royal Society of Chemistry. Reproduced with permission.^[^ ^302^ ^]^ Copyright 2015, Royal Society of Chemistry. Adapted with permission.^[^ ^303^ ^]^ Copyright 2020, Wiley‐VCH. Reproduced with permission.^[^ ^304^ ^]^ Copyright 2013, Springer Nature.

The global energy crisis and ecological degradation have highlighted the need for new generation energy products such as biofuel cells that utilize available sources like blood, sweat, or tears.^[^ ^44^ ^]^ Biofuel cells are essentially ATBs that utilize biocompatible electrochemical catalysts, including microbial, enzymatic, and non‐enzymatic types, to accelerate fuel oxidation and oxidant reduction for converting chemical energy into electricity.^[^ ^277^ ^]^ These biofuel cells are constantly re‐established by body fluids, allowing them to generate power in excess of hundreds of milliwatts over an extended period of time. Glucose is a readily available fuel in living organisms that can produce up to 16 kilowatts of electricity per gram when broken down.^[^ ^278^ ^]^ as shown in **Figure** **5**A, the byproducts of glucose breakdown, gluconolactone, carbon dioxide (CO_2_), and water, can be reinserted into the metabolism of the organism. Thus, the biofuel cell is designed to generate electricity to power IBDs using human biofuel (glucose) around the anode and biological oxidants (molecular oxygen) near the cathode. Although microbial oxidation at the anode is one of the most efficient method, large devices carrying microorganisms are not conducive for powering IBDs as ATBs. The redox processes can be described as follows:

(1) $$C_{6}H_{12}O_{6}+6H_{2}O↔6{\mathrm{CO}}_{2}+24e^{-}+24H^{+}$$

(2) $$6O_{2}+24e^{-}+24H^{+}↔12H_{2}O$$

#### 4.1.1 Enzymatic BFCs

BFCs based on enzymatic glucose oxidation have emerged as a promising alternative for generating electrical energy in living organisms. Enzymatic glucose biofuel cells (GBFCs) utilize glucose oxidase as the anode catalyst and laccase, cytochrome oxidase, or bilirubin oxidase as the cathode catalyst to extract electrical energy from the abundant glucose and oxygen present in biological fluids.^[^ ^279^ ^]^ GBFCs have been successfully implanted in various organisms, including plants (grapes and cacti), invertebrates (cockroaches, snails, clams, and lobsters), and vertebrates (rats and rabbits).^[^ ^280^, ^281^ ^]^ In particular, the specificity of the enzyme for oxygen reduction and glucose oxidation eliminates the requirement for segregation. After further development, Glucose oxidase (GOx) and glucose dehydrogenase (GDH) are the catalytic materials for the anode, while the cathode is mainly based on laccase cytochrome oxidase (COD), or bilirubin oxidase (BOD).^[^ ^282^, ^283^, ^284^ ^]^ As shown in Figure 5B_i_, An implanted GBFC with GOx as the anode and laccase as the cathode has been reported to generate electrical energy from mammalian body fluids. Implanted in the peritoneal cavity of rats, the GBFC generated an average open‐circuit voltage (V_oc_) of 0.57 V, a power density of 193.5 µW cm^−2^, and a volumetric power of 161 µW mL^−1^, making the GBFC capable of effectively powering electronic devices, such as digital thermometers.^[^ ^285^ ^]^ A GBFC with glucose and bilirubin oxidase as the anode and cathode can drive a sophisticated animal brain stimulator. The glucose and oxygen in the pigeon provide the GBFC with 0.08 mW of power, and the multi‐power management system can collect 2.84 mJ per minute, which is sufficient for neurostimulator (Figure 5B_ii_).^[^ ^133^ ^]^

However, the incomplete oxidation of glucose using enzymes leads to insufficient the lower power density and open‐circuit voltage (V_oc_) of enzyme biofuel cells making it challenging to meet the requirements of high‐power IMDs such as pacemakers. Increasing the enzyme loading area and the electroactive region area of the electrode is an effective strategy. Notable, Figure 5B_iii_ illustrates that proper structural design of bioelectrodes can improve the performance of GBFC to some extent. Enzyme/polymer composite matrix was designed with a porous structure, which improves enzyme immobilization. The resulting implantable GBFC can achieve a power density of 76.6 mW cm^−3^, which surpasses the highest reported performance by approximately 96 times.^[^ ^286^ ^]^ In addition, nanomaterials with large specific surface areas such as carbon nanotubes (CNTs) as electrodes in Figure 5B_iv_ can produce large electroactive areas that can greatly increase the loading of immobilized enzymes thus improving the performance of EBFC.^[^ ^287^ ^]^ Despite power generation efficiency limitations, GBFCs offer a viable option for generating electrical energy in vivo, with potential applications in powering electronic devices or medical implants.

#### 4.1.2 Abiotic Biofuel Cells

Natural enzymes as electrochemical catalysts face many technical bottlenecks, such as demanding environmental operability, poor stability, and unstable electron transfer, which limit the development and application of GBFCs.^[^ ^288^ ^]^ However, the development of functionalized nanomaterials in nanobiotechnology has led to the emergence of inorganic nanomaterials‐based catalysts that have relatively lower temperature and pH requirements, high thermal stability, chemical resistance, and controllable composition and structure compared to natural biocatalysts.^[^ ^289^ ^]^ For instance, nanomaterials with mimetic enzymatic activity, such as metal nanoparticles (Cu NPs, Ni NPs,^[^ ^290^ ^]^ Au NPs,^[^ ^283^ ^]^ Pt NPs^[^ ^291^ ^]^), metal oxides (Ag_2_O),^[^ ^292^ ^]^ metal sulfides (CuS,^[^ ^293^ ^]^ CdS^[^ ^294^ ^]^), and carbon compounds (Mxenes,^[^ ^295^ ^]^ MWCNTs^[^ ^296^, ^297^ ^]^), effectively decrease the volume of devices and improve the power density. The use of inorganic nanomaterials in GBFCs has led to the development of miniature BFCs that have high power densities. For example, as shown in Figure 5C_i_, a miniature BFC prepared by AuZn electrode produced power densities of 2.07 and 0.29 mW cm^−2^ in glucose solution and human serum, respectively. The microfilm fuel cell was also stable enough to operate continuously for more than 18 days and produced a potential of 0.52 V when implanted in the brains of rats.^[^ ^298^ ^]^ However, precious metal nanomaterials used as catalysts are rare and expensive, and there is a growing interest in developing inorganic nanomaterials composed of Fe,^[^ ^299^ ^]^ Ni,^[^ ^300^ ^]^ and Mn,^[^ ^301^ ^]^ which are low‐cost and biocompatible. Graphene nanocomplexes loaded with Co nanoparticles (Co/NG) can be used as both anodic and cathodic catalysts with V_oc_ of 790 mV and a considerable power density (0.15 mW cm^−2^) (Figure 5C_ii_).^[^ ^302^ ^]^ In addition, Buckypaper, carbon films, and polymers have been used to ensure the mechanical flexibility of GBFCs as ATBs. Figure 5C_iii_ gives a GBFC can be prepared by a biocatalytic Buck paper electrode modified with pyrroloquinoline quinone dependent GDH and BOD for glucose oxidation and oxygen reduction, respectively. When implanted in live gray garden slugs, the electrical energy generated by this device ranges from 2 to 10 µW.^[^ ^303^ ^]^ Porous carbon paper can be used as a support layer for the conductive anode catalyst to prepare a small size, low ohmic resistance, and low membrane stacking simulated enzyme glucose biofuel cell. This GBFC has a greatly increased loading of Pt NPs, which has enabled it to achieve a power density of 2 µW cm^−2^, as presented in Figure 5C_iv_.^[^ ^304^ ^]^

The oxidation reactions that occur in the bioanode of biofuel cells require different types of fuels such as lactic acid,^[^ ^305^, ^306^ ^]^ pyruvic acid, fatty acids,^[^ ^307^ ^]^ succinic acid,^[^ ^308^ ^]^ and amino acids^[^ ^309^ ^]^ and ascorbic acid^[^ ^310^ ^]^). Lactate is a particularly attractive biofuel due to its higher levels in body fluids, especially after intense physical activity. Lactic acid/O_2_ BFCs possess substantial theoretical energy density, attributed to their exceptional solubility and fuel energy density.^[^ ^311^ ^]^ However, its usage has been limited in vivo because of its lower content compared to glucose. Nonetheless, a sensor prepared by coupling lactate oxidase (LOx) with a ferrocene‐based redox polymer (FcMe_2_‐LPEI) as the anode, and laccase with pyrene‐anthracene‐modified carbon nanotubes (PyrAn‐MWCNT) as the cathode exhibited a V_oc_ and power density of 0.73 V and 404 µW cm^−2^, respectively.^[^ ^312^ ^]^

One of the main drawbacks of BFCs is their restricted output voltage, determined by the thermodynamic constraints imposed by the redox potential of oxygen and biofuel. Consequently, they typically exhibit an output voltage of merely 0.5 V that is inadequate for powering most IBDs (cardiac pacemakers and neurostimulators) that typically necessitate 2−3 V. Several approaches have been demonstrated to enhance the output voltage, including connecting multiple BFCs in series or storing the electricity in energy storage devices to release pulses. For example, researchers connected three clam BFCs in series parallel and stored the energy in a capacitor to power a motor.^[^ ^296^ ^]^ Similarly, the method of connecting two lobster BFCs in series was employed, which effectively increased the output voltage from 0.6 V to 1.2 V, enabling the power supply of a wristwatch for an hour.^[^ ^313^ ^]^

### 4.2 Thermoelectric Nanogenerators (TEGs) as ATBs

**Figure 6:**
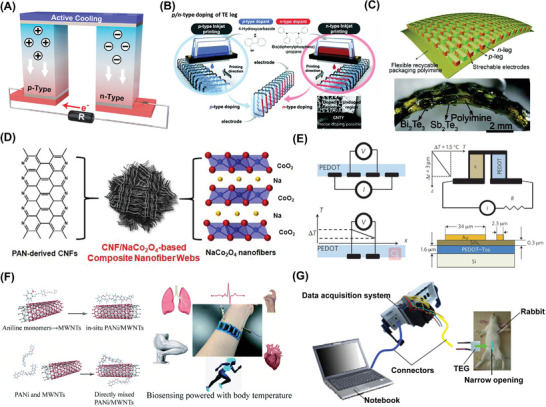
Thermoelectric Energy Harvesters (TEGs). A) The power supply mechanism of TEGs. B) Thermoelectric energy harvesting of human body heat for wearable sensors. Adapted with permission.^[^ ^317^ ^]^ Copyright 2022, Wiley‐VCH. C) TEGs based on Bi_2_Te_3_/Sb_2_Te_3_ p‐n Junctions. Reproduced with permission.^[^ ^321^ ^]^ Adapted 2021, Wiley‐VCH. D) THEs based on nano‐SiC dispersed NaCo_2_O_4_ composites. Reproduced with permission.^[^ ^322^ ^]^ Copyright 2019, World Scientific. E) TEGs based on conducting polymer (poly(3, 4‐ethylenedioxythiophene)). Reproduced with permission.^[^ ^328^ ^]^ Adapted 2011, Springer Nature. F) A gill‐mimicking thermoelectric generator based on polyaniline and multiwalled carbon nanotube. Reproduced with permission.^[^ ^332^ ^]^ Copyright 2021, Royal Society of Chemistry. G) an implantable thermal generator. Reproduced with permission.^[^ ^337^ ^]^ Copyright 2007, IOP Publishing.

TEGs based on the Seebeck effect are devices that convert a temperature gradient into a potential.^[^ ^314^ ^]^ Originally used in vehicles, factories, boilers, or other heat‐generating equipment to collect waste heat to improve the overall conversion efficiency, TEGs have also gained attention in biomedical applications due to their lightweight, reliable, maintenance‐free, and biocompatible nature for powering implanted low‐power electronic devices.^[^ ^315^, ^316^ ^]^ As shown in **Figure** **6**A, The performance of a TEG material can be evaluated by its dimensionless thermoelectric quality factor, which is defined as ZT = S^2^σT/κ, where S, σ, T, and κ refer to the Seebeck coefficient, electrical conductivity, temperature, and thermal conductivity, respectively. The maximum harvestable power of a TEG device is limited by the thermodynamic efficiency of the Carnot cycle, which is expressed as η_C_ = 1 – T_L_/T_H_ and depends on the ratio of the low and high temperatures (in Kelvin) of the device plate, represented by TL and TH, respectively. Therefore, when the temperature difference decreases, the Carnot efficiency decreases. Ideally, a maximum energy harvesting efficiency of 3.9% can be obtained with a typical body temperature (T_H_ = 310 K) and room temperature (T_L_ = 298 K). For instance, a study reported that TEGs achieve ultrahigh power factors of 4739 µW m^−1^ K^−2^ at 298 K. The TEG provides a maximum output power of 1.2 mW at ΔT = 25 K, and the heat at the hand is converted to 19.2 µW of power, which can be used to operate a red LED with the aid of a voltage boost‐up converter (Figure 6B).^[^ ^317^ ^]^

To achieve high thermoelectric performance, it is essential to have a material with high electrical conductivity to minimize electrical losses, as well as low thermal conductivity to maintain a thermal gradient and ensure continuous energy conversion. The trade‐off between thermal and electrical conductivity presents a significant challenge in the development of high‐performance thermoelectric materials.^[^ ^318^ ^]^ Nonetheless, semiconductors with low thermal conductivity hold great promise for TEG applications. These materials are highly desirable as semiconductor doping can effectively regulate their electrical conductivity without significantly impacting their thermal conductivity. This feature helps maintain a large temperature gradient across the device, which in turn results in a high thermoelectric conversion efficiency. Among these semiconductors, metallic alloys such as bismuth telluride (Bi_2_Te_3_) and antimony telluride (Sb_2_Te_3_) are n‐type and p‐type semiconductors with the highest ZT. When a temperature gradient is applied, the charge carriers inside semiconductors move from the high‐temperature end to the low‐temperature end, generating an electrical potential (Figure 6C).^[^ ^319^, ^320^, ^321^ ^]^ Besides, Figure 6D illustrates that transition metal oxides, including NaCo_2_O_4_, are another class of materials that exhibit excellent thermoelectric properties across a broad range of operating temperatures.^[^ ^322^ ^]^

However, the common bulk thermoelectric materials described above have many drawbacks for IMEs, including bulkiness, rigidity, and poor tissue fit In contrast, flexible thermoelectric materials offer the advantage of conformally contacting curved heat sources, thereby maximizing heat collection. While research has focused on conductive polymers like polyimide (PI), polyaniline, PEDOT, and polypyrrole (PPy), these materials often encounter challenges in achieving high output power density due to their low ZT values and elevated contact resistance.^[^ ^323^, ^324^, ^325^, ^326^, ^327^ ^]^ For example, the reported ZT of the PEDOT polymer system is ≈0.4, which is still poor compared to inorganic materials (Figure 6E).^[^ ^328^, ^329^ ^]^ To address this issue, researchers are exploring the use of organic/inorganic hybrid composites to enhance the electrical conductivity of the materials.^[^ ^330^, ^331^ ^]^ Incorporating carbon nanotubes and inorganic nanomaterials into polymers has been shown to enhance the power factor. Continued investigations in this field led to the advancement of high‐performance flexible thermoelectric materials that are suitable for use in IMEs (Figure 6F).^[^ ^332^, ^333^, ^334^, ^335^ ^]^

For medical implantation, achieving sufficient temperature gradient at the microscale under physiological conditions is critical for flexible TEGs. To achieve this, TEG devices are limited to being embedded only near the superficial skin layer, which limits their practical application.^[^ ^336^ ^]^ To showcase the efficacy of implanted TEGs for energy harvesting, in vitro simulations were conducted using a commercial TEG (TEC‐01706T125) attached to the surface of a pork sample. To replicate the thermal conditions of the human body, a copper sheet was positioned beneath the tissue layer and connected to a thermostatic heating device set at 37°C, while the skin surface was exposed to room temperature. This configuration generated a temperature gradient of 0.5 K, leading to an output voltage of ≈3.3 mV during the simulation. To provide additional evidence of the practicality of implanted TEGs for energy harvesting, the TEG was implanted in the abdomen of a live rabbit for 260 s, and the temperature gradient was controlled to remain at a stable 1.3 K. The output voltage reached 5 mV, and when the skin surface was covered with an ice bag, the temperature gradient increased to 5.5 K, resulting in a higher output voltage of 25 mV, as presented in Figure 6G.^[^ ^337^ ^]^

### 4.3 Devices Utilize Biopotentials as ATBs

**Figure 7:**
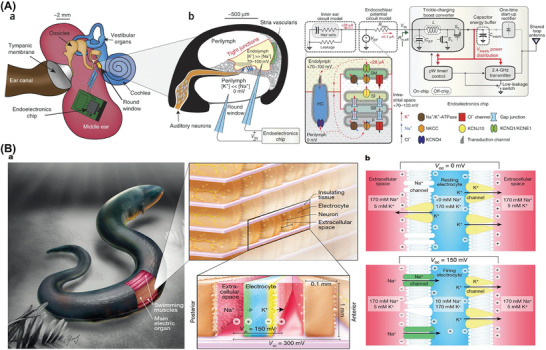
Devices Utilize Biopotentials. A) Endocochlear potential. Adapted with permission.^[^ ^30^ ^]^ Copyright 2012, Springer Nature. B) An electric‐eel‐inspired soft power source from stacked hydrogels. Reproduced with permission.^[^ ^342^ ^]^ Copyright 2017, Springer Nature.

The bioelectric potential present in the human body in many organs (heart, brain, and retina) will be another extremely valuable source of power for implantable devices in the future.^[^ ^338^ ^]^ The endocochlear potential (EP) arises from the disparity in ion concentration between the extracellular (perilymph) and the intracellular fluid (endolymph), which creates a battery‐like electrochemical gradient. In mammals, the transfer of potassium ions through ion channels, influenced by ion concentrations, generates the highest positive DC electrochemical potential ranging from 70 to 100 mV.^[^ ^339^, ^340^ ^]^ Two miniature glass electrodes were inserted into the cochlea of an anesthetized Hartley Albino guinea pig, with the electrode leads connected to an internal ultra‐low‐power wireless signal electronic chip on the external side of the guinea pig. A net positive power of 60−2840 pW was provided to load the circuit and run the chip for 5 h. Each cell of living organisms can actively or passively transport Na^+^ and K^+^ via pumps and channels to eventually generate transmembrane potentials, thus showing the unique advantages of flexibility and universality, allowing the extraction of bioenergy directly from the diverse implantation site, as shown in **Figure** **7**A.^[^ ^30^ ^]^ With further optimization of electrodes, such biopotential‐based ATBs are expected to power chemical sensors or drug transmitter actuators, facilitating the diagnosis and treatment of hearing loss and various other diseases.

In addition to endocochlear potential, other bioelectric sources have also been explored for implantable devices. For example, electrical energy is collected directly from the membrane potential of the female Xenopus laevis’ oocyte with Ag/AgCl electrodes, and the generated electrical energy is transferred through an appropriate circuit and stored in a capacitor for powering RF communications.^[^ ^341^ ^]^ The electronic organ of the electric eel is a characteristic example of a biopower source with power characteristics including a peak voltage of 600 V and a current of 1 A. The prepared stacked structured ATBs mimics the stacking of electroactive biological cells, achieving an energy density of 27 mW m^−2^ and a voltage of 110 V (Figure 7B).^[^ ^342^ ^]^ These bioelectric sources demonstrate unique advantages of flexibility and universality, allowing the extraction of bioenergy directly from diverse implantation sites. These power sources have the potential to be employed in various implantable devices, encompassing sensors and drug delivery actuators, among others.

### 4.4 Piezoelectric Nanogenerators (PENGs) as ATBs

**Figure 8:**
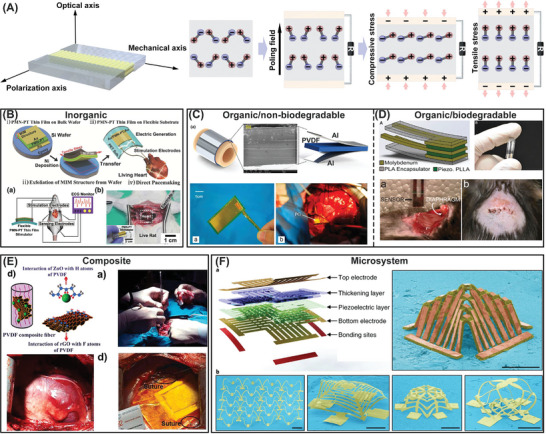
Piezoelectric nanogenerators (PENGs). A) The power supply mechanism of PENGs. B) Inorganic materials for PENGs. Adapted with permission.^[^ ^356^ ^]^ Copyright 2014, Wiley‐VCH. C) Organic and non‐biodegradable materials for PENGs. Adapted with permission.^[^ ^362^ ^]^ Copyright 2015, Elsevier. D) Organic and biodegradable materials for PENGs. Reproduced with permission.^[^ ^385^ ^]^ Copyright 2015, IEEE. E) Composite materials for PENGs. Adapted with permission.^[^ ^388^ ^]^ Copyright 2021, Elsevier. F) 3D piezoelectric microsystem of PENGs. Reproduced with permission.^[^ ^392^ ^]^ Copyright 2019, Springer Nature.

The internal organs of the human body like heart, lung, and diaphragm, are constantly engaged in a great deal of mechanical motion, and bio‐piezoelectric materials can convert the above biomechanical energy into electrical energy to power implantable biomedical devices, eliminating the drawbacks caused by battery replacement.^[^ ^90^, ^343^ ^]^ The piezoelectric effect, initially observed by the Curie brothers in 1880, involves the creation of an internal voltage difference in a material after an external force is imposed, generating an electric dipole moment in the direction of the tension of the material (**Figure** **8**A). This principle has been used to prepare PENGs that are simple in structure and have high power density, scalability, and good performance. Piezoelectric materials can be categorized into three main types: organic, inorganic, and composite piezoelectric materials include crystals such as quartz (SiO_2_), zinc oxide (ZnO), lithium niobate (LiNbO_3_), lithium germanate (LiGeO_3_) as well as ceramics like lead zirconate titanate piezoelectric ceramics (PZT), barium titanate (BaTiO_3_), and sodium bismuth titanate (BNT).^[^ ^344^, ^345^, ^346^, ^347^, ^348^, ^349^, ^350^, ^351^, ^352^, ^353^ ^]^ Inorganic piezoelectric materials have high piezoelectric coefficients and measurement accuracy but are often characterized by poor toughness, flexibility, durability, high mass, and toxicity.^[^ ^354^ ^]^

#### 4.4.1 Inorganic Materials

In 2010, the first ZnO nanowire‐based PENG was implanted in live mice to collect energy from mechanical motion (heartbeat and respiratory motion), resulting in an average voltage of 3 mV and a current of 30 pA. This groundbreaking research demonstrated the potential of PENG as a viable ATBs for powering IBDs.^[^ ^355^ ^]^ Subsequently, the electroceramic‐based PZT was developed, which has a power density of 0.29 W cm^−3^ and can provide a 2.19 V direct current to power small electronic devices, including pacemakers with frequencies below 100 Hz (Figure 8B).^[^ ^356^ ^]^ However, challenges remain in ensuring effective output power. To overcome this, (1‐x)Pb(Mg_1/3_Nb_2/3_)O_3_−xPbTiO_3_ (PMN−PT), a new generation of single crystalline materials, has been developed with a piezoelectric charge constant up to 2500 pC N^−1^. This is almost 4, 20, and 90 times higher than that of PZT, BaTiO_3_, and ZnO, respectively. The maximum output voltage and current measured in converting small biomechanical motions and mechanical deformations into electrical energy using PMN‐PT are about 8 V and 0.223 mA, respectively. These values are sufficient to power an artificial pacemaker and simulate practical applications in the rat heart.^[^ ^356^ ^]^

#### 4.4.2 Organic/Non‐Biodegradable PENGs

Organic piezoelectric materials have gained attention as a potential source of energy for biomedical devices due to their flexibility, lightweightness, and degradability in vivo. These materials include man‐made polymers such as polyvinylidene fluoride copolymer (P(VDF‐TrFE)), poly(L‐lactic acid) (PLLA), polyvinylidene fluoride (PVDF), polyvinyl chloride (PVC), etc., and bio‐piezoelectric materials with naturally anisotropic structures such as cellulose, or proteins (collagen, silk, and keratin), nucleic acids.^[^ ^357^, ^358^, ^359^, ^360^, ^361^, ^362^, ^363^, ^364^, ^365^, ^366^, ^367^, ^368^, ^369^, ^370^, ^371^, ^372^, ^373^, ^374^ ^]^

Studies have demonstrated the feasibility and effectiveness of using PVDF‐based PENGs for harvesting aortic pulsatile energy.^[^ ^362^ ^]^ In in vitro experiments, the PENGs produced a maximum output voltage (V_max_), current (I_max_), and power (P_max_) of 10.3 V, 400 nA, and 681 nW, respectively. When implanted around the aorta of a pig with a heart rate of 120 bpm and blood pressure of 160/105 mmHg, the PENG produced a Vmax and Imax of 1.5 V and 300 nA, respectively. The instantaneous output power was 30 nW, and it could charge a 1 µF capacitor to 1.0 V within 40 seconds, as shown in Figure 8C.^[^ ^362^ ^]^ PENGs that harvest energy from heartbeat motions have the potential to power a heartbeat monitoring system, eliminating the need for battery replacement and reducing secondary trauma. Additionally, using a honeycomb PDMS structure with a low elastic modulus and a PTFE film with a high charge density, a single‐layer PENG with an operating area of 10 cm^2^ and subjected to a compressive load of 10 kg c as the capability to accumulate a charge of 0.1 µC per cycle. The charge output can be amplified via a multilayer stacked structure connected in parallel.^[^ ^375^ ^]^

Generating a high voltage electrical response is a challenge for most semi‐crystalline polymers, as piezoelectricity originates in the crystalline region. However, simple electrostatic interaction can be used to assemble PENGs with sandwich structures made from negatively/positively charged fluorinated ethylene propylene and poly(vinyl alcohol), demonstrating the feasibility of ultrathin and flexible piezoelectric for epidermal and implantable electronics applications. The piezoelectric charge constant of these materials can reach 930 pC N^−1^.^[^ ^376^ ^]^ Recently, PENGs based on P(VDF‐TrFE) or P(PVDFTrFE)/PbTiO_3_ have been developed for collecting mechanical energy from the human body and converting it to electrical energy at high power levels for real‐time determination of human health factors. However, it should be noted that some of the polymers mentioned above, such as PVDF, can degrade in the body and produce the harmful substance HF.^[^ ^377^, ^378^ ^]^ Hence, further investigation is required to advance the development of biocompatible and long‐term implantable organic piezoelectric materials for use in the human body.

#### 4.4.3 Organic/Biodegradable PENGs

Biomaterials generally possess structures with high order and low symmetry, lacking inversion centers. Moreover, numerous biomolecules inherently demonstrate linear electromechanical coupling.^[^ ^379^ ^]^ Bio‐piezoelectric materials, compared with conventional inorganic and polymeric piezoelectric materials, bio‐piezoelectric materials, are biocompatible and do not produce biological contaminants after degradation in vivo, thereby avoiding the need for secondary surgical intervention.^[^ ^343^ ^]^ When based on bio‐piezoelectric materials, PENGs can degrade into non‐toxic amino acids and polysaccharides that can be reabsorbed under biologically benign or physiological conditions after completing their tasks.^[^ ^380^ ^]^ While most of these materials may exhibit a relatively low piezoelectric response compared to conventional commercial piezoelectric materials, tuning the orientation and polarization direction holds promise for improving their electromechanical coupling by several orders of magnitude.^[^ ^381^ ^]^ Thus, bio‐piezoelectric materials offer unique biodegradable properties for bioelectronics applications. For example, chitin films that have undergone ferroelectrization and controlled processing demonstrate remarkable piezoelectric properties when subjected to external mechanical stress, comparable to those of conventional fluoride materials. These chitin films exhibit a strong piezoelectric response, yielding a remarkable output current density of 177 nA cm^−2^ and exhibiting reliable durability withstanding 5000 cycles at a vertical pressure of 100 kPa.^[^ ^382^ ^]^ Silk films stretched to a maximum ratio of 2.7 before breakage showed maximum shear piezoelectric properties (−1.5 pC N^−1^) and increased II β‐phase crystallinity, making them a promising material for stretchable transducers that can ensure tissue apposition rates in living organisms.^[^ ^383^ ^]^ Repolarization of silk fibers under strong electric fields has been reported to enhance their output properties, with silk fibers polarized by a 3 × 106 V m^−1^ electric field exhibiting an arrangement of β‐sheet layers that dominated the potential (40.7 mV), three times higher than that of unpolarized fibers (13.4 mV).^[^ ^384^ ^]^ Moreover, as presented in Figure 8D, P has been successfully implanted in the abdominal cavity of mice to monitor diaphragm contraction pressure, as reported in a study.^[^ ^385^ ^]^ These findings offer exciting possibilities for integrating bio‐piezoelectric materials with tissues and organs, enabling the development of self‐sensing bionic systems in the fields of regenerative medicine, and drug delivery devices.

#### 4.4.4 Composite Materials for PENGs

To address the drawbacks of single piezoelectric materials, composite piezoelectric materials have been developed by combining organic and inorganic piezoelectric materials and through material modification and structural design (nanowires, thin films, etc.).^[^ ^386^, ^387^ ^]^ These composite materials leverage the advantages of both components, combining high flexibility and sensitivity, thereby ensuring the protection of biological organs and tissues from any potential damage. A battery‐free pacemaker has been developed using a left atrial heart energy harvester, which is a PENG. The PENG consists of a nanofiber film made of polyvinylidene fluoride‐trifluoroethylene (PVDF‐TrFE), a hybrid nanofiller made of zinc oxide (ZnO), and reduced graphene oxide (rGO). The PENG, when implanted in the body, is capable of harvesting 0.487 µJ of energy from each heartbeat, exceeding the pacing threshold energy of the heart and positioning it as a promising option for self‐powered biomedical implants (Figure 8E).^[^ ^388^ ^]^ Another notable advancement involves the development of a soft and biocompatible silk skin‐adhesive hydrogel that incorporates zinc oxide nanorods (ZnONRs). This material can generate frictional and piezoelectric energy for PENGs, which can detect mechanical motion and harvest mechanical energy. The introduction of ZnONRs improves piezoelectric performance by a factor of eight compared to pristine silk hydrogels, generating high power (1 mW cm^−2^) sufficient to activate low‐power electronic devices such as smart inhalers, oximeters, and smartwatches.^[^ ^389^ ^]^ In addition, a piezoelectric film based on 0.5Ba(Zr_0.2_Ti_0.8_)O_3_‐0.5(Ba0.7Ca_0.3_)TiO_3_ (BZT‐BCT) nanowires and PVDF has been developed as an ultrasonically‐driven nanogenerator with excellent output performance to act as a neural stimulator. This film can act as a neural stimulator, generating electrical pulses that can be remotely programmed using ultrasonic excitation, with adjustable input power and waveform.^[^ ^390^ ^]^ Finally, high‐performance and flexible nanocomposites can be synthesized by forming unique PMN‐PT nanowire layered structures on top of PDMS.^[^ ^391^ ^]^

Piezoelectric microsystems consist of piezoelectric materials that are utilized in energy conversion and medical rehabilitation. While these systems are usually planar, converting them into complex 3D frameworks can expand their modes of operation. This can be achieved through a controlled nonlinear flexural process that transforms lithographically defined 2D‐patterned electrodes and piezoelectric polymer films into 3D microsystems, opening up new application pathways. Using this method, more than twenty different 3D geometries have been created, demonstrating the versatility of the engineering approach. These structures can be tailored to produce root mean square voltages spanning from 2 mV to 790 mV, making them suitable for energy harvesting applications and for integration into biomedical devices that can be manipulated in vivo (Figure 8F).^[^ ^392^ ^]^

### 4.5 Triboelectric Nanogenerators (TENGs) as ATBs

**Figure 9:**
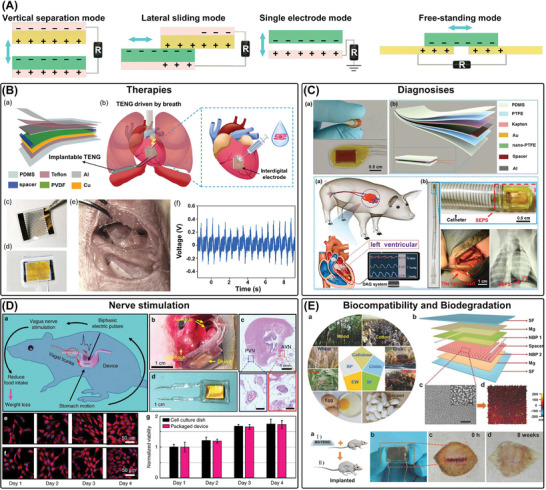
Triboelectric Energy Harvesters (TENGs). A) The power supply mechanism of TENGs. B) TENGs for treatment. Reproduced with permission.^[^ ^404^ ^]^ Copyright 2022, Elsevier. C) TENGs for detection. Adapted with permission.^[^ ^407^ ^]^ Copyright 2019, Wiley‐VCH. D) TENGs for never stimulation. Adapted with permission.^[^ ^408^ ^]^ Copyright 2018, Springer Nature. E) Biodegradable materials for TENGs. Reproduced with permission.^[^ ^416^ ^]^ Copyright 2018, Wiley‐VCH.

TENGs utilize the coupling effect of frictional initiation and electrostatic induction, and they have the advantages of low cost, wide selection of materials, universal adaptability, and flexibility. These features make them ideal for collecting low‐frequency, irregular mechanical energy from human motion and converting it to electrical energy efficiently.^[^ ^393^ ^]^ Based on the above, implantable TENGs have emerged as a promising type of ATBs. As shown in **Figure** **9**A, the contact/separation process of two insulating organic/inorganic materials creates an electric potential difference due to the gain/loss of electrons, which in turn generates opposite charges at the contact surface. To equalize the potential difference, free electrons constantly flow in both directions within the external circuit of the two materials, resulting in a pulsating current. TENG can be categorized into four types based on the operating mode, heir device structure, and external circuit linkage: vertical contact‐separation mode, contact‐sliding mode, single‐electrode mode, and freestanding triboelectric‐layer mode.^[^ ^394^ ^]^ TENGs can produce electrical energy by harnessing small movements within the body, like biological motions or microscopic tissue interactions. This capability serves as a sustainable power source for implanted electronic devices, eliminating the need for external power or batteries. Furthermore, its integrated and miniaturized design enables operation at the nanoscale, facilitating easier implantation within the body and reducing its impact on tissues.

TENGs consist of friction materials and electrode materials. The electrode materials are typically selected from metal foils, metal particles and nanowires, carbon materials (graphene, carbon nanotubes, conductive graphite), and conductive polymers (including polyaniline, PEDOT: PSS, and polypyrrole).^[^ ^395^ ^]^ Almost all materials, including metals, polymers, oxides, wood, and even human hair, and skin, exhibit frictional electrical effects, which greatly expands the scope of TENG applications.^[^ ^396^ ^]^ A diverse range of materials has been investigated for the construction of flexible TENGs, including inherently flexible polymers and thin‐film inorganic conductors. Polytetrafluoroethylene (PTFE), polyvinylidene fluoride (PVDF), fluorinated ethylene propylene (FEP), and polydimethylsiloxane (PDMS) have been used for negatively charged frictionally charged materials. Metals, synthetic polymers (polyamide (PA), polyethylene terephthalate (PET)), and natural polymers (cellulose, silk, collagen) are used for positively charged frictional materials.^[^ ^397^, ^398^, ^399^, ^400^ ^]^ Although the output power of TENGs is already superior compared to other forms of energy conversion devices, their high voltage and low current characteristics still limit their direct application to conventional electronic devices. Therefore, improving the output power of TENGs is essential for their practical application. The output performance of TENGs is significantly influenced by material properties such as surface charge density, surface roughness, and electron affinity. Surface roughness from appropriate structural designs (pyramid structure, hemispherical structure, or interlocking structure) via special preparation methods (inverted molding, spinning, or microfluidic) can improve the frictional contact area and thus obtain higher power density.^[^ ^31^, ^401^, ^402^ ^]^ Appropriate material selection, structural designs, and fabrication strategies can make TENGs a focused novel strategy for implantable mechanical energy harvesting, physiological signal detection, and intelligent treatment of diseases, with initial applications in the respiratory, neurological, cardiovascular, and digestive systems.^[^ ^403^ ^]^

#### 4.5.1 TENGs for Therapies

A TENG was successfully implanted in a living rat for the first time to extract bioenergy from its periodic breathing. The TENG consisted of a multilayer structure, including a PDMS film with a pyramidal array and an Au film as a positive/negative electrical friction layer, which allowed for precise sensing of the rat's breathing movements. During one breath, the TENG generated a V_oc_ of ≈3.73 V and a short‐circuit current (I_cs_) of 0.14 µA, which was then stored in a capacitor to power a pacemaker. The energy obtained from five respiratory movements was sufficient to regulate the heart rate of rats, representing a breakthrough in self‐driven IBDs.^[^ ^31^ ^]^ Following further research, the application possibilities of TENGs have been expanded. A TENG, prepared with PTFE and Al as negative and positive friction layers and Cu and Al as electrodes, has been used to promote the maturation of neonatal rat cardiomyocytes in vitro. Figure 9B demonstrates that the implementation of a TENG resulted in a notable upregulation of connexin 43, α‐actinin, and c‐troponin T, effectively enhancing the maturation process of neonatal rat cardiomyocytes in vitro. It has also been demonstrated that the TENG can be activated by rat respiration and rabbit heartbeat, highlighting its potential as an IMD for electrically inducing the proliferation of neonatal cardiomyocytes.^[^ ^404^ ^]^ TENGs can be considered a potential source of electrical stimulation for cell proliferation, cell migration, and alignment, and stem cell differentiation, providing higher output voltage than the cell and tissue voltage. A TENG prepared with Al and nanostructured PTFE as positive/negative electrical friction layers, respectively, had a V_oc_ of up to 65.2 V and collected energy (0.495 µJ) from each cardiac exercise cycle, significantly higher than the required endocardial pacing threshold energy (0.377 µJ). As a fully implanted symbiotic pacemaker, capable of energy harvesting and storage, as well as cardiac pacing, was developed and demonstrated successful correction of sinus arrhythmias and prevention of deterioration in large animals.^[^ ^405^ ^]^

#### 4.5.2 TENGs for Diagnosis

A multifunctional TENG is designed to provide real‐time, accurate, and continuous detection of multifarious pathological and physiological indicators. A versatile TENG has been developed for continuous monitoring of multiple physiological and pathological indicators in real‐time. The TENG incorporates a core‐shell structure, comprising a nano‐pillar PVDF film as the negative electro‐frictional layer, an aluminum foil as the positive electro‐frictional layer, and electrodes for efficient energy harvesting. It has a V_oc_ of 10 V and an I_sc_ of 4 µA when driven by small animal respiration. The multifunctional TENG is designed to provide real‐time, accurate, and continuous monitoring of various body indicators, enabling comprehensive and dynamic assessment of health conditions. One of its capabilities is the identification of, cardiomyopathy, atrial fibrillation, and arrhythmias events with 99% measurement accuracy in large animals through analysis of the peak signals. Additionally, the TENG is capable of estimating blood pressure and flow velocity by utilizing an arterial catheter. In vivo experiments conducted over two weeks demonstrate the excellent biocompatibility and durability of the material. The TENG can be integrated with a surgical catheter for minimally invasive implantation of endocardial pressure sensors, which is crucial for cardiac disease patients.^[^ ^406^ ^]^ The TENG with PTFE and a nano‐structured surface, as well as Al membranes as frictional layers, has been shown to have an increased V_oc_ from 1.2 V to 6.2 V after undergoing a surface nanostructure and corona discharge process. The TENG exhibits excellent linearity (R^2^ = 0.997) and high sensitivity (1.195 mV mm Hg^−1^) over a pressure range of 0−350 mm Hg^−1^, as demonstrated in an in vitro test system. The successful implantation of TENG in the left ventricle of an adult Yorkie pig model enabled the monitoring of intracardiac pressure and diagnosis of clinical signs such as atrial fibrillation, arrhythmia, and tachycardia (Figure 9C).^[^ ^407^ ^]^

#### 4.5.3 Neurostimulator Based on TENGs

An implant system that responds to stomach movements and regulates food intake through vagus nerve stimulation has shown promise as an effective treatment for obesity. An implant system that responds to stomach movements and regulates food intake through vagus nerve stimulation has shown promise as an effective treatment for obesity. With food intake, peristaltic movements of the stomach induce the TENGs to generate biphasic electrical signals that stimulate the vagus nerve, resulting in controlling food intake. The method was validated in a rat model, where the average body weight of the stimulated group is 38% lower than that of the control group, demonstrating the effectiveness of neurostimulation in treating obesity, as presented in Figure 9D.^[^ ^408^ ^]^

In the field of muscle function restoration, electrical stimulation has emerged as a viable treatment option. Unlike nerves, which are commonly found in small nerve bundles, muscles consist of numerous excitable motor neurons that are thinly spread throughout the muscle. To evaluate the efficacy, specificity, and adjustability of direct peripheral nerve stimulation, a water‐air TENG array was fabricated.^[^ ^409^, ^410^, ^411^, ^412^ ^]^ The intermediate suspension of thin PDMS was used to enhance the signal of TENGs by an order of magnitude in fluid under pressure. Using a flexible nerve clip interface and a TENG array with multiple pixels, researchers were able to selectively stimulate the tibial and common peroneal nerve branches in rats to control the plantar flexors and ankle dorsiflexion. Muscle activation could be controlled by applying a compression force to the interface. The effectiveness of TENGs direct nerve stimulation was demonstrated in comparison to conventional biphasic square waves.^[^ ^413^ ^]^ In addition to regulating muscle function, nerve stimulation can be utilized for the control of bladder function. These findings highlight the potential of TENG technology for direct nerve stimulation and its applications in the field of neuromuscular rehabilitation.

#### 4.5.4 Biocompatible and Biodegradable TENGs

IBDs play an important role in non‐clinical disease diagnosis and treatment for patients, yet nearly all IBDs require removal or replacement through invasive procedures. Incorporating in vivo degradable and resorbable IBDs provides a feasible approach to circumvent subsequent invasive interventions.^[^ ^53^, ^414^ ^]^ The progress in implantable TENGs based on biodegradable materials is driving the advancement of next‐generation customizable IBDs. Since the initial application of biodegradable materials in TENG, some materials have been developed and utilized for various purposes such as energy harvesting, physiological detection, and drug delivery. Researchers have further developed a fully bioresorbable natural material TENG based on bioabsorbable natural material, which is arranged into egg white (EW), silk fibroin (SF), chitin, cellulose, and rice paper (RP)) according to the triboelectric coefficient, providing a reference for selecting and structural design of future degradable TENG materials.^[^ ^54^, ^415^ ^]^ The prepared TENGs, as shown in Figure 9E, achieved a wide range of output performance with V_oc_ and I_sc_ of 8−55 V and 0.08−0.6 µA. The degradation rate of TENG was regulated by adjusting the crystallinity of filamentous by methanol, and the in vivo device failure time was 1−11 days with complete degradation in 84 days.^[^ ^416^ ^]^ In addition, a self‐powered dynamic pressure sensor with biodegradable and absorbable characteristics can convert biological pressure change into an electrical signal. The TENG exhibits exceptional sensing abilities, with a sensitivity of up to 11 mV mm Hg^−1^. In a large animal model (dog), precise tracking of pleural pressure, vascular pressure, and detection of vascular obstruction events were accomplished. Furthermore, the addition of 4% polylactic‐chitosan as a friction and adhesion layer provides superior antimicrobial ability (99%), allowing implanted TENG to be completely absorbed within 21 to 84 days after application without the risk of tissue infection.^[^ ^417^ ^]^ To extend the application of biodegradable IBDs, poly(lactic‐co‐glycolic acid) (PLGA) membranes are sealed on a support substrate of nanoporous silicon or magnesium foil to prepare a fully bioresorbable wireless data collection platform. This platform is capable of performing in body cavities and deep tissues, enabling accurate monitoring of pressure, temperature, motion, flow, thermal properties, and pH, with the potential to extend to biomolecules.^[^ ^415^ ^]^ A fully biodegradable TENG based on gelatin and electrospinning polylactic acid nanofibers achieves V_oc_ to 500 V and maximum power densities of 5 W m^−2^. The TENG exhibits excellent mechanical stability and reliability during cyclic contact and completely degrades biologically innocuous material within ≈40 days.^[^ ^418^ ^]^ Indeed, both TENGs and PENGs are dependent on mechanical motion, and insufficient motion can result in a low effective power density. Furthermore, their inherent drawback of high V_oc_ and low I_sc_ leads to an insufficient effective power supply. Moreover, when the effective area is too small, the output voltage becomes too low, making it unable to meet the requirements of high‐power IMDs.

**Table** **3** summarizes the recently developed IPHs, which have achieved in vitro energy harvesting and can be applied to power IBDs for non‐clinical disease treatment and detection. BFCs boast the advantages of autonomous powering and an extended lifespan. However, they grapple with lower energy density and the imperative consideration of biocompatibility. On the other hand, TEGs offer the benefits of multi‐source utilization and relative stability, yet they are hampered by limited efficiency and the potential need for larger volumes. PENGs, designed for miniaturization and high sensitivity, rely on external vibrations and exhibit limited energy density. Similarly designed for miniaturization, TENGs face constraints imposed by frictional forces and energy density. When navigating the landscape of implantable energy technologies, a comprehensive evaluation of the strengths and weaknesses of each technology is indispensable to meet specific application requirements and attain optimal performance.

**Table 3:** Summary of IPHs for clinical application.

Type Advantages disadvantages	Test object	Material	Application	Voltage	Power density	Ref.
**BFCs**						
Persistent and stable power supply, biocompatibility, small size; But poor stability, low energy density, high cost	−	Glucose oxidase (GOx)/bilirubin oxidase (BOD)	implantable bioelectronic and wearable devices	2.3 V	60 µW cm^−2^	[419]
	−	Glucose dehydrogenase (GDH)‐multiwalled carbon nanotube (MWCNT)/BOD‐MWCNT	implantable bioelectronic and wearable devices	0.56 V	13.5 µW cm^−2^	[420]
	−	GOx‐(MgO‐templated mesoporous carbon) (MgOC)/BOD‐MgOC	−	0.94 V	180 µW cm^−2^	[421]
	Rat	Carbon nanotube (CNT)‐GOx/CNT‐laccase	Brain	0.95 V	1.3 mW cm^−2^	[422]
	Rabbit	MWCNT‐GOx/MWCNT‐laccase	IBDs	0.65 V	16 µW cm^−3^	[423]
	Rat	Au‐Au nanoparticles	Brain	0.4 V	2 µW cm^−2^	[424]
	−	Pt‐rGO/FeCo‐Ketjen black	−	−	−	[425]
	Slug	PQQ‐dependent glucose dehydrogenase (PQQ‐GDH)/ bilirubin oxidase (BOx)	−	0.6 ± 0.01 V	875 ± 5 µW cm^−3^	[303]
	Human	Lactate oxidase (LOx)‐carbon/BOD‐carbon	Skin	3.66 V	−	[426]
**TEGs**						
Persistent and stable power supply, biocompatibility,	Human	Bismuth telluride (Bi₂Te₃)	Skin	14.8 mV	20 µW cm^−2^	[427]
	Human	Polyaniline (PANi)/MWCNT	Skin	1.1 mV	−	[332]
high energy density; But large volume, poor stability, high cost	Human	SWCNT/PVA/PEI	−	2 mV	−	[428]
	Rabbit	TEC1‐01706T125	Cecum	1.2 V	−	[337]
**Biopotentials**						
Miniature size, long lifespan, incompatibility; But Low power output, limited range, and durability, complex design	Pig	Glass microelectrodes	Inner ear	30–55 mV	−	[30]
	Frog	Intracellular electrodes	Xenopus oocytes	90 mV	−	[341]
	−	Polyacrylamide hydrogel	Active contact lens	110 V	27 mW m^−2^	[342]
**PENGs**						
Miniature size, long lifespan, incompatibility; But Low power output, limited range, and durability, complex design	Bovine	PZT	Pacemaker	4.32 V	0.18 µW cm^−2^	[349]
	Pig	PVDF	Blood pressure monitoring	2 V	−	[429]
	Rat	PVDF/modifiedTiO_3_@dopamine	Neurostimulator	13.5 V	0.16 mW cm^−2^	[390]
	Porcine	Fluorinated ethylene propylene (FEP)/ poly(vinylalcohol) (PVA)	Skin and subcutaneous tissue	3 V	1.2 W cm^−2^	[376]
	Rat	Poly‐L‐lactide (PLLA)	Abdominal cavity	0.9 V	−	[385]
	Human	Silk/ZnO nanorods	skin	12.5 V	1 mW cm^−2^	[389]
**TENGs**						
Long lifespan, high energy conversion efficiency, small size; But Limited power output, limited by environment, complex design	Pig	Kapton‐Al	Heart rate monitoring	14 V	−	[430]
	Pig	PTFE‐Al	Neurostimulator	10 V	−	[406]
	Rat, dog	Poly(lactic acid)‐(chitosan 4%)/Mg	Respiratory and blood pressure monitoring	3.2 V	−	[417]
	Rat	Poly(L‐lactide‐co‐glycolide) (PLGA)/Mg	subdermal dorsal region	40 V	32.6 mW cm^−2^	[415]
	Rabbit	Poly(ε‐caprolactone)‐polydimethylsiloxane/collagen aggregate‐chitosan	Cartilage therapy	25 V	67.5 mW m^−2^	[431]
	Rat	Nitrile/silicone rubber	Muscle and nerve stimulator	20 V	−	[432]

## 5 External Wireless Power Transfer (WPT) Systems as ATBs

Implantable bioelectronic systems for biomedical applications are becoming increasingly miniaturized and available for long‐term use, leading to a growing demand for ATBs.^[^ ^433^ ^]^ Nevertheless, the replacement of depleted batteries in integrated implants becomes necessary, especially when they occupy a significant portion of the total volume. Ideally, ATBs should be a non‐invasive solution applicable to all types of tissues.^[^ ^9^ ^]^ To address this, wireless power transfer (WPT) has emerged as a promising solution for driving advanced multifunctional microelectronic devices. WPT is a technology that allows for the transmission of energy from a power source to a load without the need for physical wires or direct electrical connections.^[^ ^434^ ^]^ At present, WPT technologies for IBDs include radio frequency (RF), ultrasonic, and photovoltaic technologies. These technologies are beneficial for constructing completely wireless, lightweight, miniaturized, and battery‐free permanent implantable systems or extending the life of ATBs by providing external energy where possible.^[^ ^433^, ^435^ ^]^ However, designing WPT systems requires careful consideration of crucial parameters, including the system size, the distance between the external environment and the IBDs, transmission efficiency, operating frequency, and power consumption to ensure tissue safety.

### 5.1 RF Energy Harvesters (RFs) as ATBs

**Figure 10:**
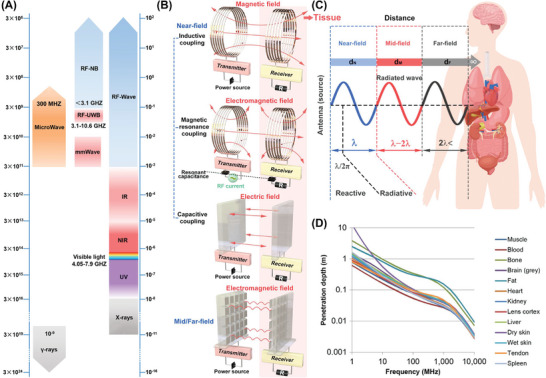
A) Electromagnetic spectrum. B) The power supply mechanism of WPT. C) Classification of electromagnetic waves according to their distance from the source. D) The penetration depth and attenuation level of RF waves with different frequencies in different human tissues. Reproduced with permission.^[^ ^442^ ^]^ Copyright 2016, Society for reproduction and fertility.

**Figure** **10**A illustrates the common electromagnetic waves used in WPT, including visible light (400−700 nm), near‐infrared light (0.7−10 µm), and radio frequency radiation (1 mm−100 km). An RF energy harvester is a type of ATBs that captures RF radiation (magnetic or electric field) emitted by the transmitter (coil or metal plate) via a receiver (coil or metal plate) implanted in the body. The captured energy is then converted to DC via a rectifier circuit to power IBDs. The typical operating frequencies of RF energy harvesters range from MHz to GHz, and output voltages of tens of volts are achieved by optimizing the geometric design of the transmitter and receiver to provide sufficient power for a variety of IBDs. RF energy harvesting can be divided into three main categories based on the relative distance between the energy transmitter and receiver compared to the wavelength of electromagnetic waves as illustrated in Figure 10B,C: Near‐field wireless energy transmission (capacitive coupling (CC), inductive coupling (IC), magnetic resonance coupling (MRC)) use inductive fields that do not emit outward and are widely used to transfer energy over short distances (mm−cm, < λ), with operating frequencies typically in the range of Hz−MHz. In the reactive near‐field region, energy does not propagate in a conventional manner but instead oscillates back and forth between the energy source and the surrounding field. The behavior of energy in the reactive near‐field region is complex and can be described as the interference between the electromagnetic field and the energy source. This interference leads to intricate energy patterns and interactions between the source and the surrounding field. It's only when the electromagnetic field reaches the radiative near‐field region that energy begins to radiate and propagate outward in the form of radiation into the far‐field region.^[^ ^436^, ^437^ ^]^ If the distance between the transmitter and the receiver is less than the wavelength being emitted, near‐field technology is typically chosen, utilizing the coupling of magnetic or electric fields for power transmission. Due to the rapid decay of near‐field behavior with increasing distance from the source, special attention is required in the design to ensure that the receiver is positioned as close to the source as possible, aiming for a high coupling ratio and maximal transmission efficiency. In the air, this distance is usually only a few centimeters. The application scope of this near‐field technology is relatively limited, primarily confined to situations where devices need to be placed near the skin, such as in the case of subcutaneous medical implants. Therefore, optimizing the layout of the electromagnetic field to ensure reliable energy transmission within a limited distance is a crucial consideration in such applications.^[^ ^438^ ^]^ Mid‐field wireless energy transmission is an emerging technology in wireless power supply for IBDs, which is used for systems with a separation distance of approximately one wavelength between the transmitter and receiver (cm−m, λ−2λ).^[^ ^439^ ^]^ For systems utilizing mid‐field technology, the transmitter can be designed with high directivity, making it crucial to position the receiver accurately for maximizing energy transmission, as the required power is greater than in near‐field scenarios. Far‐field wireless energy transmission (> m, > 2λ, MHz−GHz/THz) uses radiated fields radiated outward by electromagnetic waves (microwave radiation and laser).^[^ ^440^ ^]^ Devices relying on far‐field technology require more power compared to mid‐field technology. However, there are advantages and disadvantages to different energy transmission methods.

IC and MRC have high power transmission efficiency and large transmission distances, while microwave radiation and laser types impose the highest impact on biosafety. The MRC type has an increased impact on biosafety as the operating frequency increases. The safety level of human exposure to RF radiation is considered for the frequency range of 3−100 GHz. As per the IEEE standard, the basic limit for local permissible exposure is set at 2 W kg^−1^ for a specific absorption rate averaged over 10 g of tissue. Considering the safety concerns associated with long‐term RF exposure, radiated wireless charging for IBDs typically operates in the low‐power range.^[^ ^439^, ^441^ ^]^ The stability of energy transmission efficiency is determined by several factors, including the relative orientation of the antenna pair, external electromagnetic interference, standing waves generated by reflections from environmental obstacles, absorption associated with body fluids or tissues, and heat generation by human tissue. Additionally, the penetration depth and energy dissipation of RF waves in different tissues vary with frequency and the tissue's dielectric properties.^[^ ^414^ ^]^ For instance, low‐frequency RF waves have longer wavelengths and correspondingly deeper penetration depth, as shown in Figure 10D.^[^ ^442^ ^]^ However, a larger antenna size is also required for the wave to oscillate, which is necessary for achieving optimal performance. Therefore, selecting the appropriate RF wave frequency and antenna size should be based on the specific needs of the application and consider the characteristics of different frequencies and antenna sizes. The actual operating distance of the energy harvesting system is limited to 0.1−3 m, highlighting the importance of proper design for implantable devices.

Miniaturized, flexible inductively coupled or RF energy harvesting devices commonly use islands of active electronic components, serpentine interconnected bridges, mesh structures, or multilayer stacked structures.^[^ ^443^ ^]^ Biodegradability is a key property of ATBs for power harvesters. To achieve biodegradability, various materials can be utilized such as silicon nanomembranes (Si NMs) for semiconductors, soluble metals like magnesium (Mg), zinc (Zn), iron (Fe), and molybdenum (Mo) for interconnects, as well as silicon dioxide (SiO_2_) and magnesium oxide (MgO) for dielectrics. For substrates, options include polycaprolactone (PCL), polylactic acid (PLA), and poly(lactic‐co‐glycolic acid) (PLGA).^[^ ^444^, ^445^, ^446^ ^]^

#### 5.1.1 Near‐Field Energy Transfer

**Figure 11:**
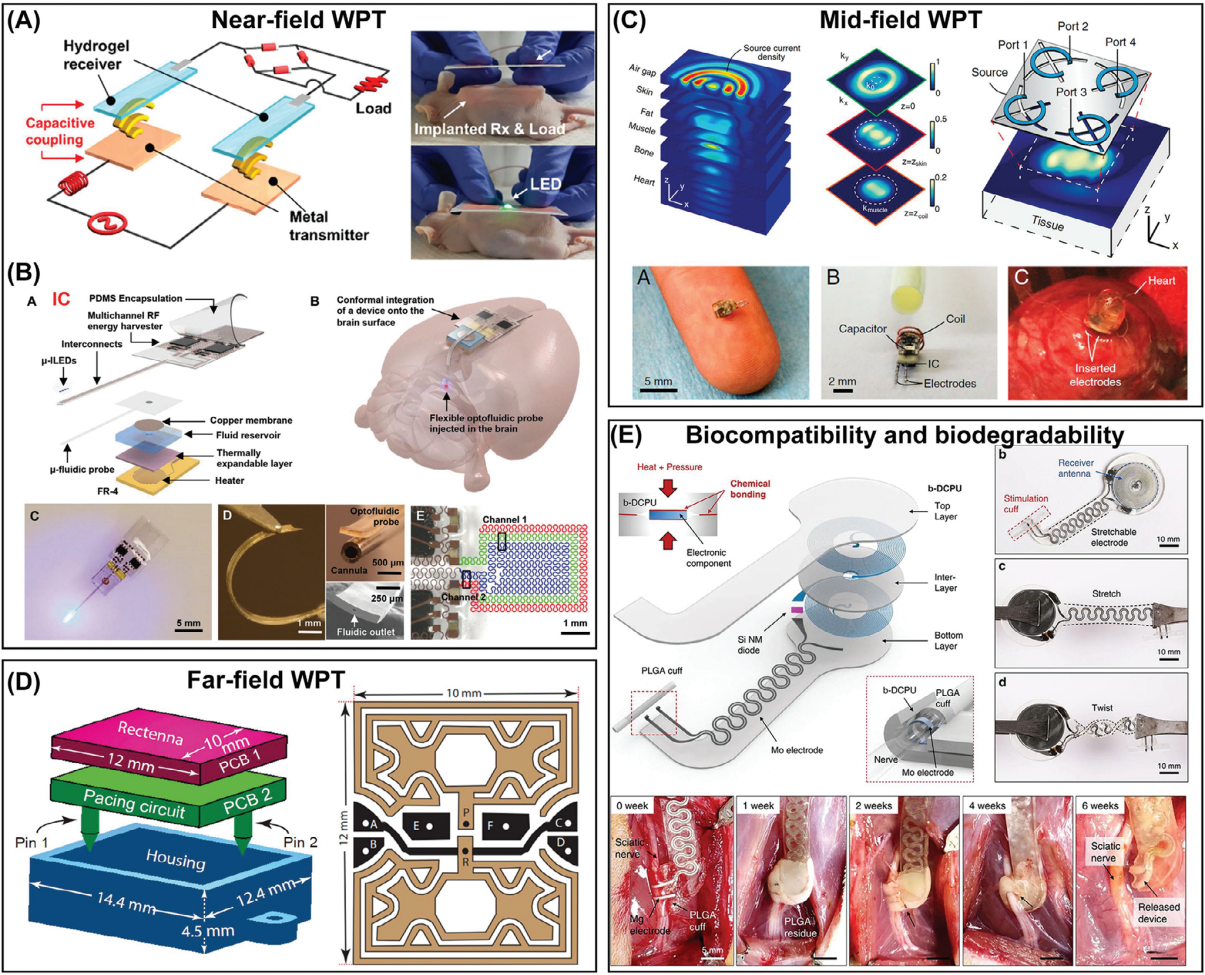
A) An implantable ionic wireless power near‐field transfer system (CC). Reproduced with permission.^[^ ^447^ ^]^ Copyright 2020, American Chemical Society. B) A miniaturized, battery‐free photofluid system based on IC with wireless pharmacology and optogenetics potential. Reproduced with permission.^[^ ^448^ ^]^ Copyright 2018, Wiley‐VCH. C) Wireless power transfer to deep‐tissue microimplants based on Mid‐field. Reproduced with permission.^[^ ^48^ ^]^ Copyright 2014, National Acad Sciences. D) A novel RF‐powered wireless pacemaker and a wearable transmit‐antenna array based on Far‐field WPT. Reproduced with permission.^[^ ^453^ ^]^ Copyright 2018, IEEE. E) Bioresorbable electronic stimulators. Reproduced with permission.^[^ ^455^ ^]^ Copyright 2020, Springer Nature.

The Ionic Wireless Power Transfer is a cutting‐edge near‐field wireless power transmission system that relies on CC principles to deliver a current of 4 mA. The system comprises a biocompatible ionic hydrogel receiver and a metal plate transmitter, which work together to enable power transfer. The coupling mechanism underwent a thorough analysis, and the device was tested on an implantable device, demonstrating its ability to transmit power through the skin while maintaining the input signal amplitude below 10 V for safety purposes. To assess the effectiveness as a power source for implantable devices, experiments were conducted by inserting the system into the subcutaneous area of a mouse, and power was transmitted through the skin to illuminate an implanted LED. These results showcase the remarkable ability to transmit power wirelessly and its potential for powering future implantable devices (**Figure** **11**A).^[^ ^447^ ^]^ Miniaturization of ATBs is a crucial characteristic for various biomedical applications, especially when combined with softness. In this context, a battery‐free wireless soft optofluidic system was developed, weighing only 220 mg and measuring 125 mm^3^. This miniaturized device provides wireless drug delivery and optogenetic stimulation, allowing spatiotemporal control of targeted neural circuits in behaviorally free animals. The system comprises a soft microfluidic probe, a micron‐scale LED array, a snake‐structured stretchable multichannel wireless RF energy harvester, and other auxiliary equipments. The device is free from bulky batteries and provides independent control of both light and fluid delivery, enabling efficient and reliable operation. The IC energy harvester employs two independent channels operating at peak power transfer efficiencies (1.8 and 2.9 GHz). When implanted in the mouse's head, the IBDs continuously collect the transmitted wireless RF energy, ensuring unrestricted motion and robust operation, as illustrated in Figure 11B.^[^ ^448^ ^]^ The potential of ATBs based on MRC principles for applications with minimal impact on subject behavior is greatly enhanced by their high integration, miniaturization, and flexibility. A wireless, battery‐free, fully implantable multi‐peak recording and neuromodulation tool has been developed that integrates miniaturized dual optogenetic probes, a multimodal optogenetic stimulator, a thermal imaging device, and an energy management system guided by behavior. This device was successfully implanted subcutaneously in small finches, allowing for optical modulation of their song.^[^ ^449^ ^]^ The convergence of battery‐powered and battery‐free technologies presents a comprehensive solution for implantable systems, enabling reliable power supply and allowing for seamless whole‐body implantation. It has the potential to facilitate the exploration of brain function and therapy of various degenerative diseases, including defibrillators, deep brain stimulators, drug pumps, and cardiac pacemakers.^[^ ^450^ ^]^ Powerful customizable controls using off‐the‐shelf smartphones will greatly facilitate therapeutic interventions.

#### 5.1.2 Mid‐Field Energy Transfer

The miniaturization of mid‐field WPT sources presents a significant challenge. While wireless powering has been achieved, transferring energy beyond the surface depth of the tissue has been limited by the use of large coils, which are not suitable for miniaturized implants. However, this limitation can be overcome by adopting a mid‐field powered approach, where a patterned metal plate is used to induce spatially limited and adapted energy transfer through the propagation pattern in the tissue.^[^ ^451^, ^452^ ^]^ This approach allows for the powering of a miniature implant (measuring only 2 mm and weighing 70 mg) that provides remote wireless monitoring and control of the heart and is significantly smaller than a conventional cardiac pacemaker. The levels of exposure remain below safe thresholds for human health, and the micro‐implant can be powered at milliwatt levels to deliver multiple physiological stimuli to deep tissues, even at depths greater than 5 cm (Figure 11C).^[^ ^48^ ^]^

#### 5.1.3 Far‐Field Energy Transfer

There has been a notable surge in interest surrounding wearable and implantable devices that utilize far‐field energy tramsfer strategies. For instance, a leadless pacing system that relies on RF‐powered technology features a wearable transmitting antenna array and an implantable silicon rectifier antenna. This system incorporates a fractal planar dipole antenna as a receiver, matched to a modified rectifier circuit. Figure 11D gives an efficient transmitter array based on RT/Duroid 6010 and it is designed to achieve a directional beam of the antenna in the ground plane. The system was successfully validated in a sheep in vivo model and achieved a power transfer efficiency of 65% at an appropriate operating frequency (954 MHz).^[^ ^453^ ^]^ Abdi and Aliakbarian also conducted a study on far‐field RF‐powered leadless pacemakers, which used a silicon rectified antenna system consisting of a circularly polarized (CP) helical antenna and a single diode detector based on a Schottky diode. The system demonstrated a 40% efficiency in converting RF power to direct current power, generating a direct current voltage of 0.2 V. These results were obtained when the system was exposed to an input power level of 20 dBm and tested using a three‐layer tissue model.^[^ ^454^ ^]^

#### 5.1.4 Biocompatible and Biodegradable WPTs

Improved materials, device structure, and integration strategies can enable the development of an implantable bioresorbable electrical stimulation platform using soft, elastomechanical, and long‐life materials. As shown in Figure 11E, a traumatic peripheral nerve injury requires an electrical stimulation platform comprising a wireless receiver (RF collector), a stretchable Mo electrode with a filamentous serpentine interconnect structure, and a PLGA stimulation cuff. In a mouse model, a 30‐day electrical stimulation treatment using a stimulation cuff wrapped around the sciatic nerve and an RF collector unit inserted subcutaneously effectively alleviated muscle atrophy due to denervation. The bioresorbable polyurethane packaging ensured the trouble‐free and reliable operation of the implantable electrical stimulator for over one month, surpassing the recovery period associated with traumatic nerve injury.^[^ ^455^ ^]^ Jahyun Koo et al. developed a fully bioresorbable electronic system drug release control system triggered by electrochemical etching that combines a biocompatible wireless power collection function with an electrochemically degradable valve and reservoir system composed of a bioresorbable polymer. the RF power collector generates a super‐electric potential that causes the gate opening to be electrochemically etched. Magnesium electrodes with PBTPA shell form the polymer reservoir, magnesium coil collector, silicon nanofilm diode, parallel plate capacitor in a magnesium^−1^/silicon dioxide/magnesium multilayer stack, and Mg metal gate valve. To regulate levels of glucose in the blood, implantable drug delivery systems that are bioresorbable and contain three reservoirs of drugs that can be independently controlled were subcutaneously implanted in rats.^[^ ^456^ ^]^

### 5.2 Ultrasound‐Induced Energy Harvesters (UEHs) as ATBs

**Figure 12:**
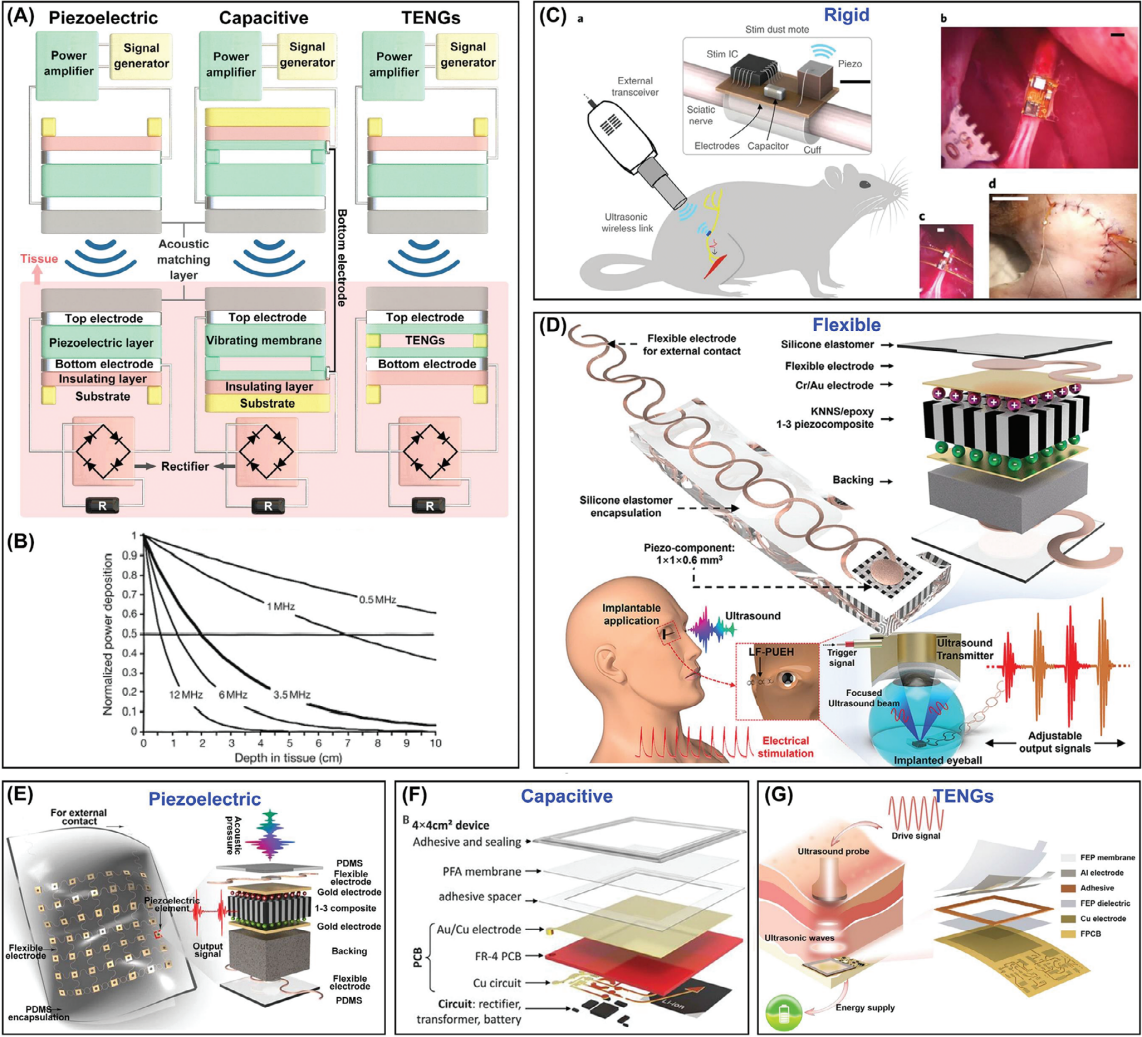
Ultrasound‐induced energy harvesters (UEHs). A) The power supply mechanism of UEHs. B) The relationship between normalized power attenuation of sound waves and tissue depth and ultrasound frequency. Reproduced with permission.^[^ ^470^ ^]^ Copyright 2014, Elsevier. C) A miniature rigid neural stimulator with ultrasonically powered bidirectional communication. Reproduced with permission.^[^ ^464^ ^]^ Copyright 2020, Springer Nature. D) Ultrasound‐induced flexible wireless energy harvesting for potential retinal electrical stimulation application. Reproduced with permission.^[^ ^137^ ^]^ Copyright 2019, Wiley‐VCH. E) Flexible piezoelectric ultrasonic energy harvester array for bio‐implantable wireless generator. Reproduced with permission.^[^ ^467^ ^]^ Copyright 2019, Elsevier. F) Transcutaneous ultrasound energy harvesting using capacitive triboelectric technology. Reproduced with permission.^[^ ^37^ ^]^ Copyright 2019, Science. G) Transcutaneous ultrasound energy harvesting using TENGs. Reproduced with permission.^[^ ^459^ ^]^ Copyright 2022, Cell.

UEHs offer a promising method for wireless energy transmission as ATBs to power IBDs. The mechanism of ultrasound‐induced energy transmission can be divided into two types: piezoelectric, capacitive, and TENGs, as shown in **Figure** **12**A. Piezoelectric UEH systems are based on the piezoelectric‐semiconductor coupling effect, while capacitive UEH systems are usually attributed to electret electrostatic and frictional electrical energy harvesting. The current focus of research is on developing flexible piezoelectric ultrasound energy transmission devices to achieve better adaptability to non‐planar tissue and organ surfaces, as well as to improve ultrasound energy conversion efficiency and output power. This technology utilizes ultrasound vibration waves transmitted to a transducer, which converts the vibrations into electrical energy, enabling the implanted device to be powered without the need for additional surgery and minimizing the risk of surgical infections. With the increased complexity and functionality of implantable medical devices, power consumption has also increased, making the use of ultrasound a promising solution for energy supply. Ultrasound offers advantages such as high penetration, low in vivo attenuation, concentrated energy, no electromagnetic interference, good directionality, and larger transmission distance, enabling deeper implantation depths and smaller size receivers for implantable devices.^[^ ^50^ ^]^ Furthermore, ultrasound frequency selection is not restricted by national regulations and can be safely used in the human body at certain intensities, with less tissue heating when operating at lower frequencies.^[^ ^137^ ^]^ However, further exploration is needed to ensure safe and effective energy solutions for implantable medical devices and their clinical applications through transcutaneous energy delivery.

While ultrasound waves experience lower attenuation in the body compared to electromagnetic waves, their power transfer efficiency can still be affected by factors such as reflection and standing wave formation. In the case of standing waves occurring due to reflection from transmitter and receiver surfaces, the power transfer efficiency of sound waves is impacted. One approach to address this issue is demonstrated by the broadband piezoelectric ultrasonic energy harvester (PUEH), which modifies the input ultrasonic frequency when standing wave formation occurs.^[^ ^457^ ^]^ Figure 12B illustrates the attenuation curve depicting the changes in ultrasound wave intensity with varying frequencies as they propagate through tissue. Low‐frequency waves can penetrate deeper into the tissue, as shown by the 0.5 MHz wave that can still retain most of its power after traveling 10 cm in tissue, while the 12 MHz wave loses 80% of its power after traveling only 1 cm.

The thickness of the UEHs devices is directly related to the operating frequency and the transducer should operate in resonance to achieve optimal efficiency. However, reducing the device thickness to the micron range is challenging due to the significant increase in tissue attenuation at higher frequencies. Additionally, to avoid tissue overheating and cavitation, the maximum power output must be strictly regulated, and the FDA limits the acceptable ultrasound intensity for biomedical diagnostic applications to 720 mW cm^−2^.^[^ ^50^, ^458^ ^]^ UEH systems can be categorized into three types based on their energy conversion mechanism: piezoelectric, capacitive, and TENG. Piezoelectric ultrasound energy harvesters (PUEHs) systems rely on the piezoelectric‐semiconductor coupling effect, while capacitive ultrasound energy harvesters (CUEHs) and TENG systems are typically based on electret electrostatic and frictional electrical energy harvesting (TUEHs).^[^ ^457^, ^459^ ^]^ Flexible implantable PUEHs are extensively researched using a variety of membrane compositions. These compositions include piezoelectric composites such as PZT and ZnO, as well as epoxy resins. Interconnects such as Cu and Au are also used, along with substrates and packaging materials such as polyimide (PI), polydimethylsiloxane (PDMS), and poly(parylene)‐C.^[^ ^460^, ^461^ ^]^ Traditional bio‐implantable piezoelectric ultrasound energy harvesters have predominantly utilized plate structures due to their superior theoretical acoustic power output. However, diaphragm structures have emerged as an alternative option, providing advantages such as conformal contact with non‐planar surfaces of human tissues, as well as enhanced energy generation capabilities compared to plate structures.^[^ ^462^, ^463^ ^]^

#### 5.2.1 Rigid UEHs

IBDs that are clinically approved are limited by battery requirements and large size compared to the corresponding tissue. Researchers have been working to miniaturize ATBs to address these limitations. One promising approach involves the development of wireless, lead‐free, and battery‐free implantable neurostimulators.^[^ ^458^ ^]^ These devices have dimensions of only 1.7 mm^3^ and comprise a PUEH, an electricity storage capacitor, and functional connectivity achieved through an integrated circuit. An ultrasonic link and a handheld external transceiver are utilized to provide power and enable bidirectional communication with the stimulator. The integrated circuit demonstrates efficient collection of ultrasound power in vitro porcine tissue, decodes downlink data for stimulation parameters, and generates current‐controlled stimulation pulses. When the device is implanted acutely into the sciatic nerve of anesthetized rats, it generates consistent stimulation across various physiological responses (Figure 12C).^[^ ^464^ ^]^ Another notable advancement is the introduction of neural dust, a wireless and scalable ultrasound backscatter system designed to power and communicate with IBDs. In a study by Dongjin Seo, it was demonstrated that ultrasound can effectively power millimeter‐scale devices in tissue, allowing for the reliable transmission of electromyogram and e‐neurogram signals from anesthetized rats. These findings provide strong evidence for the potential of ultrasound‐based neural interface systems to significantly advance the field of bioelectronic‐based disease therapies.^[^ ^465^ ^]^

#### 5.2.2 Flexible UEHs

However, the aforementioned rigid devices lack soft attachment to corresponding tissues, reducing the comfort of the subject, so the development of flexible devices is necessary. A flexible, minimally invasive system for monitoring tissue oxygen levels has been developed, consisting of a piezoelectric crystal PZT (750 × 750 × 750 µm^3^), µLED, oxygen sensing membrane, and optical filter assembled and encapsulated with poly(parylene)‐C. All components are integrated on a flexible plate, resulting in a final implant with a volume of 3 × 4.5 × 1.2 mm^3^ and a detection volume of approximately 0.26 mm^3^. This study showcases the capability of the IBDs to collect continuous and real‐time data at depths up to several centimeters in both sheep and isolated porcine tissue. The results provide evidence supporting the potential of these devices for monitoring deep tissue conditions in clinical applications, including surgery and intensive care.^[^ ^466^ ^]^ In addition, environmentally friendly, non‐biotoxic, lead‐free piezoelectric composites have been considered for the preparation of millimeter‐scale flexible ultrasonic wireless energy harvesting patches. These patches convert acoustic energy to electricity through piezoelectric effects and can be mounted on complex surfaces. Improved block‐cutting and filling techniques were used to fabricate the microstructure of the piezoelectric composites, yielding improved electrical and acoustic properties. In ex vivo experiments in an implanted environment, considerable current signals above the average threshold for retinal stimulation (e.g., currents > 72 µA and current densities > 9.2 nA µm^−2^) were obtained for retinal electrical stimulation to restore vision in people with neurodegenerative diseases, demonstrating the great potential for integration for electrical stimulation applications on implantable biomedical devices, as presented in Figure 12D.^[^ ^137^ ^]^

#### 5.2.3 UEHs Based on Different Principles

Efficient energy harvesting from weak ultrasonic pressure fields is an important area of research. One approach involves using a multilayer piezoelectric electret with strain‐enhanced piezoelectricity. By introducing an array of stomata connected in parallel in a dielectric layer that is sandwiched between a pair of electrets, the device is capable of providing a significant peak output power of ≈13.13 mW and an I_sc_ of ≈2.2 mA when implanted 5−10 mm below tissue. This is above the power and current thresholds required for IBDs and neural stimulation, respectively.^[^ ^463^ ^]^ Moreover, the feasibility of powering an implantable bioelectronic device and using it as a neuroprotein for peripheral nerve stimulation has been demonstrated. One strategy for generating higher conversion efficiency is to design and fabricate flexible PUEH arrays by integrating a large mass of piezoelectric pixels in an elastomeric membrane. These arrays have the capability to produce voltage outputs of up to 2 Vpp and current outputs surpassing 4 µA, which can be applied to IBDs for therapy and monitoring (Figure 12E).^[^ ^467^ ^]^ Another technology for harvesting ultrasound that can compete with piezoelectric effects in vivo and power IBDs is the capacitive triboelectric electret. Figure 12F gives a thin, implantable vibrating friction generator that uses ultrasound to transfer mechanical energy through the skin, and fluid is powered by ultrasound‐induced displacement of a PFA polymer film on the micron scale to generate electrical energy by contact charging. The device charges a lithium‐ion battery in water at a rate of 166 mC s^−1^ and can produce voltages and currents of 2.4 V and 156 mA under pig tissue.^[^ ^37^ ^]^ Based on the charge separation effect generated by the friction between nano materials, a nano friction generator can produce electricity by utilizing the friction between nano materials induced by ultrasonic vibration, thus showing the potential application in ultrasonic energy transmission. As shown in Figure 12G, a highly efficient and flexible nano friction generator with scalability has been applied in vivo in pigs and mice, demonstrating an available energy density (0.23 mW cm^−2^).^[^ ^459^ ^]^ However, due to the small scale of the nano friction generator, the energy density is relatively low and may not meet the demands of some high‐power applications. Moreover, the performance and stability of nano materials may be influenced by environmental conditions, which need further improvement for long‐term and reliable applications in complex environments.^[^ ^468^ ^]^

To achieve the biocompatible and biodegradable properties of ATBs, researchers are exploring new materials with biocompatibility, biodegradability, and bioactivity, new fabrication techniques, and structural designs. One promising development is the MXene hydrogel ATBs, which mimic tissue and convert ultrasound power into electrical energy using simple structures. Unlike current implantable UEHs, the M‐gel ATBs utilize the flow vibration potential, an electroacoustic phenomenon, as its underlying principle. Additionally, the output power of the ATBs can be enhanced by electrically coupling it with frictional bands. This generator has the potential to rapidly charge electronic devices buried under a centimeter thick beef, making it an ideal power source for implantable devices.^[^ ^469^ ^]^ To enhance the efficiency of energy harvesting, researchers are actively exploring new mechanisms as well as combinations of existing ones. This includes investigating thermoelectric, magnetic, bioenergetic, photoacoustic, and optoelectronic principles, either individually or in combination, to develop more efficient and versatile energy harvesting technologies. By exploring these different mechanisms and their synergies, researchers aim to maximize energy capture and conversion for various applications in the field of bioelectronic devices.

### 5.3 Photovoltaic Devices (PVs) as ATBs

**Figure 13:**
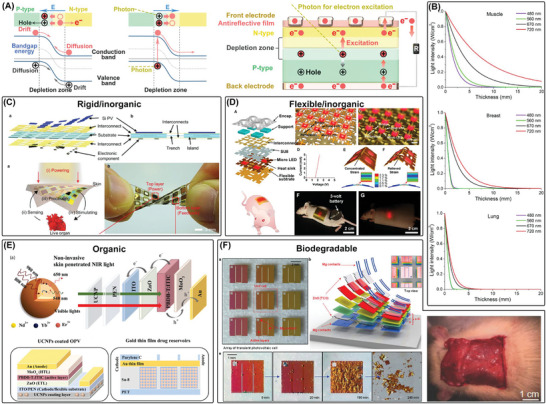
Photovoltaic Devices (PVs). A) The power supply mechanism of PVs. B) Attenuation of visible and near‐infrared light in different human tissues. Reproduced with permission.^[^ ^480^ ^]^ Copyright 2016, Springer Nature. C) Rigid and inorganic materials for PVs. Adapted with permission.^[^ ^482^ ^]^ Copyright 2021, Wiley‐VCH. D) Flexible and inorganic materials for PVs. Reproduced with permission.^[^ ^484^ ^]^ Copyright 2020, National Acad Sciences. E) Organic materials for PVs. Reproduced with permission.^[^ ^485^ ^]^ Copyright 2021, Elsevier. F) Biodegradable PVs. Reproduced with permission.^[^ ^490^ ^]^ Copyright 2015, Royal Society of Chemistry.

PVs utilize the photovoltaic effect to capture photons and generate current and voltage through p‐n junction diodes. As shown in **Figure** **13**A, when a p‐n junction, formed by the combination of p‐type and n‐type semiconductor materials, is exposed to light, photons are absorbed and converted into electron‐hole pairs. Electrons are excited to higher energy levels and flow from the p‐type region to the n‐type region, generating electric current because of the potential difference between the two regions. This conversion of light energy to electrical energy is achieved through the photovoltaic effect, which occurs within the semiconductor material. The bandgap energy of the semiconductor material determines the voltage produced by the solar cell. It is important that the bandgap energy is smaller than the energy of the incident light to allow electrons to jump from the valence band to the conduction band. Photovoltaic (PV) cells demonstrate high efficiency in converting light in the near‐infrared spectral region (700−2500 nm), with an external quantum efficiency approaching 100%.^[^ ^471^ ^]^ This characteristic makes them an attractive choice for efficient solar cells, with the added advantage that conventional PVs can function in their environment and harness solar energy without requiring additional optical modules. In biomedical applications, PV microcells are frequently encapsulated within soft living tissues such as skin, fat, muscle, bone, and even deeper tissues.^[^ ^472^ ^]^

PVs can be divided into two main categories based on the materials: crystalline and non‐crystalline materials. The most commonly used materials for crystalline PVs are amorphous silicon, microcrystalline silicon (µ c‐Si), monocrystalline silicon (c‐Si), and polycrystalline silicon.^[^ ^473^ ^]^ Amorphous PV cells are typically less costly and more flexible than crystalline materials, although slightly less efficient. Gallium is a soft metal material that is often combined with compounds such as arsenic, nitrogen, and phosphorus. GaAs, GaInP, and GaP have higher thermal stability, and GaP and GaAs are commonly used in flexible thin‐film photovoltaic cells with breakdown voltages much higher than silicon's breakdown voltage of 200 V.^[^ ^474^, ^475^ ^]^ These materials usually have better power generation efficiency in the NIR region due to their higher bandgap and lower reverse saturation current, while amorphous silicon (a‐Si) is more suitable for visible light. In addition to the material selection, the transmittance of the encapsulation material is also an important fundamental property for PV cells to efficiently utilize light in the NIR range. Conventional encapsulation materials such as ceramics, metals, SiO_2_, SiC, Al_2_O_3_, and diamond are incompatible with the manufacturing process and soft tissue due to their hardness, and unfavorable to water vapor penetration.^[^ ^476^, ^477^ ^]^ Therefore, new encapsulation materials such as polyimide (PI), PDMS, and liquid crystals have been investigated to preserve the flexibility and biocompatibility of PV cells while ensuring their transmittance.^[^ ^478^, ^479^ ^]^ The attenuation and absorption of visible light in tissue present significant challenges, as shown in Figure 13B, with a penetration depth typically less than 2 cm. Therefore, the use of photovoltaic technology for ATBs is often limited to areas of low optical attenuation in tissue, such as in the retina or superficial subcutaneous regions.^[^ ^480^ ^]^

#### 5.3.1 Inorganic/Rigid PVs

Back in 1999, Murakawa et al. proposed a wireless photovoltaic (PV) system operating in the near‐infrared (NIR) spectrum (810 nm) for powering cardiac pacemakers.^[^ ^481^ ^]^ Since then, the development of PV cells as a wireless power solution for implantable medical devices, such as pacemakers, neurological devices, and retinal prostheses, has been widely investigated. As shown in Figure 13C, In an implanted light‐driven device, a Si PV, electronics, elastomer substrate (PDMS), snake wire (Au/Ti = 300/200 nm), and PI package are included. The PV has a maximum output power of 4.74 mW and a total area of 0.49 cm^2^. The flexible double‐sided design of the device reduces the necessary area by 50%, thereby creating additional space for enhanced power generation and the integration of more intricate functional circuits. This versatile form enables the device to accommodate higher power output and more complex functionalities. The flexible double‐sided feedback implant is well‐suited for powering smaller medical devices that require sustainable power, such as cardiac pacemakers (requiring ≈100 µW), cardiac defibrillators (requiring ≈500 µW), and drug pumps (requiring ≈1 mW).^[^ ^482^ ^]^ Increasing the energy transfer power is an important topic, and one strategy is to prepare flexible PV arrays. By integrating Si PV, NIR receivers, and LEDs on a flexible PI film with metal traces, a flexible wireless optogenetic system for light driving and light control was realized. Experiments conducted both in vivo and in vitro have demonstrated that the wireless transmission of power and remote control to the optogenetic device through skin tissue is feasible, with a sufficient power output of ≈10.3 mW. To achieve more advanced behavioral control studies on freely moving animals in experimental sites of different sizes and shapes, additional enhancements are required, such as tailored miniature LEDs for particular animal models and the integration of more effective photovoltaic devices to minimize device size.^[^ ^483^ ^]^

#### 5.3.2 Inorganic/Flexible PVs

Kim and colleagues have recently developed a wireless photonic power system that combines a flexible array of GaAs as a subcutaneous NIR light collector and a flexible array of µLEDs as an ex vivo NIR light source, as presented in Figure 13D. This complete system is capable of providing in vivo output power of 8.2 W (effective area of 0.11 cm^−2^) to drive a pulsed stimulator implanted in mice, and can also charge additional power (5.9 W) from the embedded microbattery.^[^ ^484^ ^]^ Kwangsun Song has devised an innovative approach that utilizes ultrathin double‐junction solar microcell arrays with a size of 760 µm × 760 µm and a thickness of 5.7 µm made of GaInP/GaAs. These arrays are printed on flexible polyimide films and connected with sputtered metal (Ti: 30 nm/Au: 300 nm) for circuitry. This design is characterized by its multiple layers of biocompatible transparent polymers, including SU‐8, Norland Optical Adhesive, and PDMS. This simple yet effective design allows for a thin and flexible structure, improved mechanical compatibility with the skin, and enhanced durability. Additionally, the energy output of the implant can be easily modified by adjusting the interconnections or adding more solar microcells, thus enabling control over parameters such as Isc, Voc, and output power.^[^ ^149^ ^]^

#### 5.3.3 Organic PVs

Thin film organic photovoltaic (OPV) technology has been explored in various applications. In one study, an OPV was prepared using a polyethylene naphthalate (PEN)/ITO/ZnO/PBDB‐T: ITIC/MoOx/Au layer‐stacked structure. When exposed to near‐infrared light, this system generated a current pulse that triggered the dissolution of a gold film, leading to the release of a drug from the system. This approach enabled precise, controlled release of drug reservoirs for weeks or months, depending on the design.^[^ ^485^ ^]^ In another study, researchers demonstrated the integration of an organic electron ion pump (OEIP) with OPV on flexible parylene‐C substrates for local and on‐demand delivery of pharmacologically active substances, such as alkali metal ions and neurotransmitters (Figure 13E). The researchers used metal‐free phthalocyanine (H_2_Pc) and N,N'‐dimethylperylene‐3,4,9,10‐tetracarboxylic diimide (PTCDI) to create organic thin‐film bilayer photovoltaic pixels arranged in series and/or vertical series to provide the voltage of 2.5−4.5 V required to operate a high‐resistance electrophoretic ion pump.^[^ ^485^ ^]^ Finally, in a separate study, researchers used OPVs to power a red LED on top of the skin, with the LED being driven by light emitted from a 1.5 cm thick finger.^[^ ^486^ ^]^

#### 5.3.4 Biocompatible and Biodegradable PVs

Implantable PVs have shown promising potential in various biomedical applications. However, their non‐biodegradable nature limits their usage in bioresorbable electronics and temporary medical implants. To address this issue, researchers have developed a bioresorbable monocrystalline silicon PV microcell that uses biocompatible and biodegradable materials for all its components, including the active layer, electrodes, interconnects, and encapsulation.^[^ ^487^, ^488^ ^]^ When exposed to 1 solar light, the microcell generates ≈64 W of electrical power and a V_oc_ of 4.25 V, which can drive a rat subcutaneous blue light‐emitting diode under 4 mm thick fat‐bearing pig skin. After completing the task, the entire PV system completely dissolved in the subscapular region of mice after 4 months in vivo, with no inflammatory response in the surrounding tissues observed during histological analysis. This technology offers great potential for powering bioresorbable electronic implants.^[^ ^489^ ^]^ Another approach to creating fully degradable PV devices is the use of amorphous silicon (a‐Si) transient thin‐film solar ATBs. Kang et al. reported a degradable a‐Si solar ATBs, which utilized a ZnO and Mg electrode layer. This electrode layer exhibited a dissolution rate of a few hours, followed by the complete dissolution of the a‐Si film within a few days due to the disparate hydrolytic properties. Figure 13F illustrates the dissolution behavior and biocompatibility of different materials, including Si, amorphous silicon a‐Si, polycrystalline silicon (poly‐Si), germanium (Ge), and germanium alloys. This research opens up exciting opportunities for creating fully degradable PV devices for biomedical applications.^[^ ^490^ ^]^

**Table** **4** summarizes the recently developed ATBs, which have achieved in vitro energy harvesting and can be applied to power IBDs for non‐clinical disease treatment and detection. RFs offer the advantages of contactless operation and all‐weather charging. However, their efficiency is influenced by distance and signal strength, and susceptibility to electromagnetic interference is a potential drawback. UEHs are suitable for deep tissues and relatively stable, but they exhibit lower energy density, and device design is constrained by ultrasonic propagation characteristics. In comparison, PVs have the benefits of high energy density and wireless charging. Nevertheless, their performance is contingent on lighting conditions, and their positioning is constrained during implantation.

**Table 4:** Summary of external WPT system for clinical application.

Type Advantages disadvantages	Test object	Application	Material	Frequency or wavelength	Distance	Power	Power density	Operation region	Ref.
**RFs**									
Non‐invasive, controlled energy transfer; But limited power transfer efficiency, low anti‐interference, limited frequency	Rat	Nerve stimulator	−	1 MHz	10 mm	127 mW	40.4 mW^−2^	Near‐field	[491]
	Pig	Drug release		40 MHz	15 mm	19 dbm	−	Near‐field	[492]
	Pig	Pacemaker	−	13.56 and 40.68MHz	11, 8.5 cm	0.3, 0.8 W	−	Near‐field	[493]
	Pig	Electrostimulation devices	−	1.6 GHz	> 5 cm	195 µW, 200 µW (heart, brain)	−	Mid‐field	[48]
	Rabbit	Ingestible electronics	−	1.2 GHz	−	173 µW	374 µW cm^−2^	Mid‐field	[494]
	Pig	Pacemaker	−	1.6 GHz.	> 4 cm	216mW	42 dB	Mid‐field	[495]
	Rabbit	IOP monitoring	−	3 GHz	5 cm	300 mW	4.69 W cm^−3^	Far‐field	[496]
**UEHs**									
High energy density, compact size; But limited power transmission, limited penetration depth	Human	Cardiac pacemakers	EBR systems	313−385 kHz.	5.3−22.5 cm	12.86 ± 15.70 mJ	93.1 mW cm^−2^	−	[497]
	Pig, rat	IBDs	PZT	40 kHz	1.85−50 cm	100 µW	304 mW cm^−2^	−	[498]
	−	IBDs	Nylon	20−120 kHz	3 mm	−	−	−	[468]
	Pig, rat	IBDs	FEP/Al	28 kHz	4 cm	> 10 mW	0.23 mW cm^−2^		[459]
	Human	Cardiac pacing	WiCS‐LV system	−	10 cm	−	−	−	[499]
	Pig	Subcutaneous space	PZT	1 MHz	10–15 mm	300 mW	−	−	[500]
**PVs**									
Renewable energy, high energy density, low maintenance; But limited power output, photorestricted, high cost	Rat	Biodegradable IBDs	Silicon	780 nm	2 mm	60 µW	560 µW cm^−2^	NIR	[489]
	Rat	IBDs	GaAs	850 nm	4−15 mm	0.21–9.53 µW	29–1224 µW cm^−2^	Sunlight, NIR	[472]
	Rat	Pacemaker	GaInP/GaAs	Standard solar spectrum (AM 1.5 g)	539–675 µm	647 µW	10 mW cm^−2^	Sunlight	[149]
	Rat	Electrocardiogram (ECG)	Silicon	Standard solar spectrum (AM 1.5 g)	−	5.6 mW	−	Sunlight	[482]
	−	Drug delivery systems	Upconversion nanoparticles	808 nm	−	−	−	NIR	[485]

## 6 Challenges and Outlooks

### 6.1 Flexibility and Integration Tactics

Advances in materials science, engineering technology, and micro‐ and nano‐fabrication techniques have made it possible to develop highly flexible, efficient, and biocompatible ATBs for intelligent precision medicine. However, the weight, size, and rigidity of conventional batteries still pose significant challenges to the practical implantation of highly integrated flexible circuits into tissues. To ensure a small size and high output performance, it is crucial to develop ATBs with high integration, output efficiency, and energy conversion rates, as well as ultra‐thin and flexible biosafe materials and novel multidimensional structures. Integration must also consider changes in implant site dynamics, impact on tissue, and energy dissipation. Establishing widely accepted standards for practical applications and industrialization is essential. These standards should include defined output metrics of ATBs, such as maximum output power, internal resistance, energy conversion efficiency, energy transfer efficiency, and stability. To quantify the performance of tissue‐engineered generators, Zi et al. introduced value figures in 2015.^[^ ^501^ ^]^

ATBs that avoid irritation and foreign body reactions can be prepared using flexible materials and tissue scaffold strategies for their components, including electrodes, electrolytes, septa, and receivers. These strategies ensure that the output performance is maintained while retaining lightness, integration, and flexibility, allowing the device to adapt to the mechanical properties and shape of the implant site and withstand undesired tissue compression and bioerosion over long periods. The flexibility of other functional modules, such as energy management systems, sensors, signal processing and control units, and wireless communication units, is also expected to be further developed in the manufacturing of fully flexible integrated devices. To ensure reliable connections between these functional modules, circuits formed by materials such as gold, platinum, copper, or carbon nanomaterials can be used for wire or sputtering. These connection circuits should have long‐term biochemical, geometric, and mechanical stability and provide smooth and reliable signal and power transmission.

### 6.2 Power Management

Effective power management is crucial for improving the efficiency of energy harvesting and reducing energy dissipation. Typically, two primary energy consumption patterns exist, each necessitating specific power management strategies:

(1) *Storage of the Generated Energy*: The energy generated by the implantable device near a moving organ, such as the heart, lungs, or arterial blood vessels, is usually in the form of periodic pulses. Therefore, alternating current needs to be converted to direct current and reserved in an energy storage device, such as rechargeable batteries or supercapacitors. However, these devices typically have high impedance (≈8−10 MΩ), which may result in significant energy loss due to impedance mismatch during energy transfer. To address this issue, researchers have proposed various methods. For example, Niu et al. introduced a two‐stage power management circuit for TENG, comprising two automated electronic circuits and a coupled inductor. This approach achieved a board efficiency of 90% and an overall efficiency of 60%, which is equivalent to two orders of magnitude of direct charging.^[^ ^502^ ^]^ Another study proposed a novel switched‐capacitor DC‐DC converter for external wireless energy transfer, which generates dual output voltages simultaneously for implantable electronics and provides four‐phase reconfigurable logic for boost and buck conversion. This approach achieved a conversion efficiency of 85.26% for a monolithic integrated photovoltaic power supply circuit. These findings represent significant progress toward the development of truly autonomous implantable electronics.^[^ ^503^ ^]^

Whether utilizing IPHs or WPT charging methods, the majority of implantable electronic devices necessitate an energy storage component, typically employing chemical batteries and supercapacitors. Rechargeable batteries, capable of enduring multiple charge‐discharge cycles, boast a significantly higher energy density compared to disposable batteries, rendering them a prevalent choice. However, despite their popularity, they still entail surgical removal. As a result, the trend in future development leans toward degradable and biocompatible batteries. Nevertheless, achieving controlled degradation and recyclable charging performance poses ongoing challenges, particularly when juxtaposed with supercapacitors. Studies, exemplified by those conducted by Lan et al., underscore the viability of supercapacitors as energy storage devices facilitating energy transfer between devices and electrical appliances.^[^ ^254^ ^]^

(2) *Directly Powered IBDs*: Single or composite energy storage systems (batteries and ultracapacitors), biofuel cells, and cochlear potentials can directly power IBDs due to their relatively stable output. However, power management methods are needed to avoid unnecessary power consumption or to provide sufficient voltage for specific tissues. Typically, lithium‐ion batteries are inadequate as a power source for left ventricular assist devices or total artificial hearts that require a current of 0.5−3 A and voltage of 20−30 V. One solution is to connect multiple cells in series to form a battery pack and use a battery management system (BMS) for multiplier capability and safety protection testing. Biofuel cells are another option for maintaining continuous output pulses from pacemakers, but their low output voltage (< 1 V) often does not meet the voltage requirements of most pacemakers (2−3 V). To increase the output voltage of biofuel cells, charge pumps, and DC‐DC converters are commonly employed. These devices efficiently step up the low voltage output of the biofuel cells to higher voltage levels, allowing for compatibility with various electronic systems and applications. Another solution is the development of hybrid harvesting systems to harvest energy from multiple sources, such as an RF energy harvester and an integrated power supply for biofuel cells and ultracapacitors. This approach can offset any limitations imposed by the lack of energy from one source and ensure energy output for IBDs even under exceptional conditions where one or more energy sources are not available.

### 6.3 Biological Safety

Biosafety is a critical consideration for IBDs, and it is important to standardize their evaluation. Absolute biosafety encompasses several factors, including biocompatibility of the ATBs, safety of the implantation procedure, modest biodegradability, and in vivo designable durability. Biocompatibility refers to the ability of implant materials to come into contact with biological systems without causing any adverse biological effects. This includes factors such as chemical composition, response to biological materials, and mechanical origins. Biodegradable materials are designed to undergo decomposition in physiological solutions within a specific timeframe or at a controlled rate. These materials break down into smaller components through natural processes, such as enzymatic reactions, hydrolysis, or metabolic pathways. On the other hand, bioresorbable materials are those that can be absorbed or excreted from the body without causing harm or undesirable effects on cell proliferation. These materials are carefully engineered to ensure compatibility with the body's natural processes, allowing for their gradual absorption or elimination without triggering inflammation or adverse reactions. The distinction between biodegradable and bioresorbable materials lies in their degradation mechanisms and the desired outcome in terms of their fate within the body.

To date, only a few implanted electronics have undergone long‐term in vivo testing. The longest test was conducted on rabbits for 7 months, while the shortest was conducted on rats only for 5 hours. The encapsulation plays a crucial role in protecting the bioelectrode components of the device and maintaining the activity of the ATBs by preventing interference from body fluids or biomolecules. By enclosing the ATBs and their components within a protective barrier, material leakage is prevented, ensuring the integrity of the device. The functional lifetime of the implanted electronic device can be designed according to the degradability of the encapsulation material. However, for implanted electronics to be routinely used in humans, they must exhibit excellent cycling performance in large mammals tested over a lifetime. This requires addressing three key issues: (1) designing rational ATBs for power and reducing mechanical or biochemical dissipation, (2) screening the right location in the implant space, and (3) reducing negative side effects on organs.

Clinical standards for implantable electronics are challenging to establish due to scientific, engineering, and medical ethical considerations. Nevertheless, it is critical to establish these standards in compliance with specifications established by professional organizations such as CE (Conformité Européenne), FDA (Food and Drug Administration), ISO (International Organization for Standardization), and other regulatory bodies.

## 7 Conclusions

Here, we comprehensively introduce the technology of ATBs for powering biomedical electronic devices, covering their working mechanism, material selection, structural design, output performances, biosafety, and experimental applications. We classify ATBs into three categories based on their operating characteristics and energy sources: energy storage devices, energy harvesters from the human body, and WPT technology from the external environment. We also highlight several common limitations and challenges of power supply methods, including the coordination of high‐power output and miniaturized integration, improved energy conversion efficiency, flexibility, and bio‐safety, and further we provide insights into the development trends of various drive technologies. The fundamental bottleneck and contradiction in miniaturizing ATBs while maintaining their power output and voltage is their energy conversion efficiency. For instance, smaller sizes often lead to lower output power and voltage for chemical batteries. Hence, screening superior base materials or innovative structures becomes needful according to different application scenarios and working life, which is essential for practical applications. Additionally, it is critical to lucubrate the durability and degradability of promising energy harvesters synergistically. Moreover, there is an urgent need to establish evaluation criteria to qualitatively and quantitatively assess the performances of various ATBs and their potential for practical applications and commercialization, while controlling their mass manufacturing costs. Very recently, IBDs such as neurostimulators, cardiac pacemakers, cardiovascular sensors, and drug delivery pumps have been rapidly evolving. To achieve effective disease prevention, diagnostic assessment, real‐time tracking, and precision intervention, it is critical to store, harvest, and transfer energy for ATBs. Although challenging, ATBs remain an important enabler for personalized, all‐encompassing smart medicine and intelligent health detection.

## Conflict of Interest

The authors declare no conflict of interest.
